# Characterization of a Large Group of Individuals with Huntington Disease and Their Relatives Enrolled in the COHORT Study

**DOI:** 10.1371/journal.pone.0029522

**Published:** 2012-02-16

**Authors:** E. Ray Dorsey, the Huntington Study Group COHORT Investigators

**Affiliations:** Department of Neurology, Johns Hopkins Medicine, Baltimore, Maryland, United States of America; University of Florida, United States of America

## Abstract

**Background:**

Careful characterization of the phenotype and genotype of Huntington disease (HD) can foster better understanding of the condition.

**Methods:**

We conducted a cohort study in the United States, Canada, and Australia of members of families affected by HD. We collected demographic and clinical data, conducted the Unified Huntington's Disease Rating Scale and Mini-Mental State Examination, and determined *Huntingtin* trinucleotide CAG repeat length. We report primarily on cross-sectional baseline data from this recently completed prospective, longitudinal, observational study.

**Results:**

As of December 31, 2009, 2,318 individuals enrolled; of these, 1,985 (85.6%) were classified into six analysis groups. Three groups had expanded CAG alleles (36 repeats or more): individuals with clinically diagnosed HD [n = 930], and clinically unaffected first-degree relatives who had previously pursued [n = 248] or not pursued [n = 112] predictive DNA testing. Three groups lacked expanded alleles: first-degree relatives who had previously pursued [n = 41] or not pursued [n = 224] genetic testing, and spouses and caregivers [n = 430]. Baseline mean performance differed across groups in all motor, behavioral, cognitive, and functional measures (p<0.001). Clinically unaffected individuals with expanded alleles weighed less (76.0 vs. 79.6 kg; p = 0.01) and had lower cognitive scores (28.5 vs. 29.1 on the Mini Mental State Examination; p = 0.008) than individuals without expanded alleles. The frequency of “high normal” repeat lengths (27 to 35) was 2.5% and repeat lengths associated with reduced penetrance (36 to 39) was 2.7%.

**Conclusion:**

Baseline analysis of COHORT study participants revealed differences that emerge prior to clinical diagnosis. Longitudinal investigation of this cohort will further characterize the natural history of HD and genetic and biological modifiers.

**Trial Registration:**

Clinicaltrials.gov NCT00313495

## Introduction

Huntington disease (HD) is an autosomal dominant neurodegenerative disorder resulting from an unstable expansion of a cytosine-adenine-guanine (CAG) trinucleotide repeat in the *Huntingtin* gene [1]. The CAG repeat length normally varies from 6 to 35 CAG units. Repeat lengths from 27 to 35 are considered “high normal” and may expand in subsequent generations [2]–[4]. Repeat lengths from 36 to 39 exhibit reduced penetrance, with disease manifestations occurring at a later age or not at all [3]–[5]. Alleles with forty or more repeats are fully penetrant and inevitably associated with neuronal degeneration and the progressive motor, cognitive, and behavioral features of HD [6]. Although longer CAG repeat expansions are associated with earlier disease manifestation [7]–[9], age of onset varies considerably for any given CAG repeat expansion [10].

The prevalence of HD is approximately 1 per 10,000 individuals, with a significant population at risk for the disease [11]–[12]. Improved understanding of and treatments for HD may, therefore, benefit not only those with manifest disease but also asymptomatic individuals who carry an expanded allele. Few treatments are available that specifically target HD, and no therapies currently prevent or delay disease onset or progression. We, therefore, conducted a cohort study of affected, unaffected, at-risk, and not at-risk individuals from the HD community to characterize the natural history of HD by collecting clinical data and biological samples to enhance the design of future clinical trials aimed at reducing the burden of HD. We report the baseline characteristics of the study population.

## Methods

The protocol for this trial and is available as supporting information; see Protocol S1

### Study design

The Cooperative Huntington Observational Research Trial (COHORT) is an observational study designed to collect phenotypic data and biological samples from individuals with HD and their family members.

### Setting

Beginning on February 14, 2006, investigators at 44 sites in the United States (n = 38), Canada (n = 4), and Australia (n = 2) enrolled research participants. We report baseline data collected through December 31, 2009. The study was concluded on June 30, 2011.

### Participants

Eligible research participants were from four groups: (1) individuals with clinically diagnosed HD, (2) individuals who pursued genetic testing prior to baseline, carry an expanded allele, but did not have clinically diagnosed HD; (3) first-degree or second-degree relatives of individuals in the first two groups; and (4) spouses or caregivers of individuals enrolled from group one or two. Individuals under 18 years could only enroll if they were clinically diagnosed with HD.

### Ethics

The institutional review board of the University of Rochester and each site approved the protocol. All study participants provided written informed consent, or, if unable had an authorized representative provide consent on their behalf. Participants agreed to baseline and annual evaluations for an indefinite time period with no predetermined limit on the sample size. To protect the confidentiality and data of participants, all were assigned a unique identification number without identifying information.

### Outcomes

At baseline, a site investigator and coordinator obtained demographic and clinical data, performed a complete physical and neurological exam, including the Unified Huntington's Disease Rating Scale (UHDRS) [13] and Mini Mental State Examination (MMSE) [14], and collected a blood sample for DNA isolation and for establishing an optional transformed lymphoblastoid cell line. At follow-up visits, new clinical events and current medications were recorded and an examination, including the UHDRS and the MMSE, was performed. Individuals reporting scores above a pre-specified threshold for depressed mood or suicidal ideation were referred to a mental health professional.

### *Huntingtin* CAG repeat genotyping

The *Huntingtin* CAG repeat size was determined by polymerase chain reaction amplification, using genomic DNA extracted from blood and, if provided, lymphoblastoid cell lines [15]. All genotyping was performed at a single site (Center for Human Genetic Research, Massachusetts General Hospital, Boston, Massachusetts). Alleles with 36 or more repeats were considered expanded. Individual genotypes remained anonymous and were not communicated to any party.

### Reportable events

To promote the safety of participants, a clinical monitor and an independent event monitoring committee evaluated reportable events, including suicides, suicide attempts, deaths other than suicides, and premature withdrawals.

### Optional assessments

Participants had the option to provide blood to generate a lymphoblastoid cell line for future research, to be informed of clinical trials, and to complete a baseline Family History Questionnaire (which is not included in this report) that was updated annually to track births, deaths, and HD diagnoses.

### Statistical methods

Based on prior genetic testing results, *Huntingtin* genotyping, and baseline clinical diagnosis, participants were classified into six groups. An affirmative response to UHDRS question 80, “Based on the entire UHDRS, do you believe with a confidence level ≥99% that this subject has manifest HD?” classified the individual as having clinically diagnosed HD.

Three groups had an expanded allele: individuals with clinically diagnosed HD, first-degree relatives who had pursued genetic testing, and first-degree relatives who had not pursued genetic testing. Three groups did not have an expanded allele: first-degree relatives who had pursued genetic testing, first-degree relatives who had not pursued genetic testing, and spouses and caregivers.

Participants' demographic, clinical, and genetic characteristics were compared across the six groups using descriptive statistics. For each characteristic, an overall test of heterogeneity of means or proportions was performed. To provide some protection against multiplicity effects, additional comparisons were restricted to those characteristics for which the overall test was significant. For these characteristics, additional testing was limited to six comparisons: (1) clinically diagnosed HD vs. all other groups; (2) clinically diagnosed HD vs. relatives with expanded alleles; (3) clinically diagnosed HD vs. spouses and caregivers; (4) relatives with an expanded allele who had pursued genetic testing vs. those who had not pursued genetic testing; (5) relatives with expanded alleles vs. relatives without an expanded allele; and (6) relatives without an expanded allele who had not pursued genetic testing vs. those that had pursued genetic testing. Continuous outcomes, adjusted for age and gender, were compared using analysis of covariance models that were used to conduct overall tests of heterogeneity of means, estimate contrasts of the group means, and evaluate the six comparisons. Categorical outcomes were compared using chi-square or Fisher's exact tests. Hypothesis testing was conducted at the two-sided significance level of 5%.

## Results

### Participants

**Figure 1:**
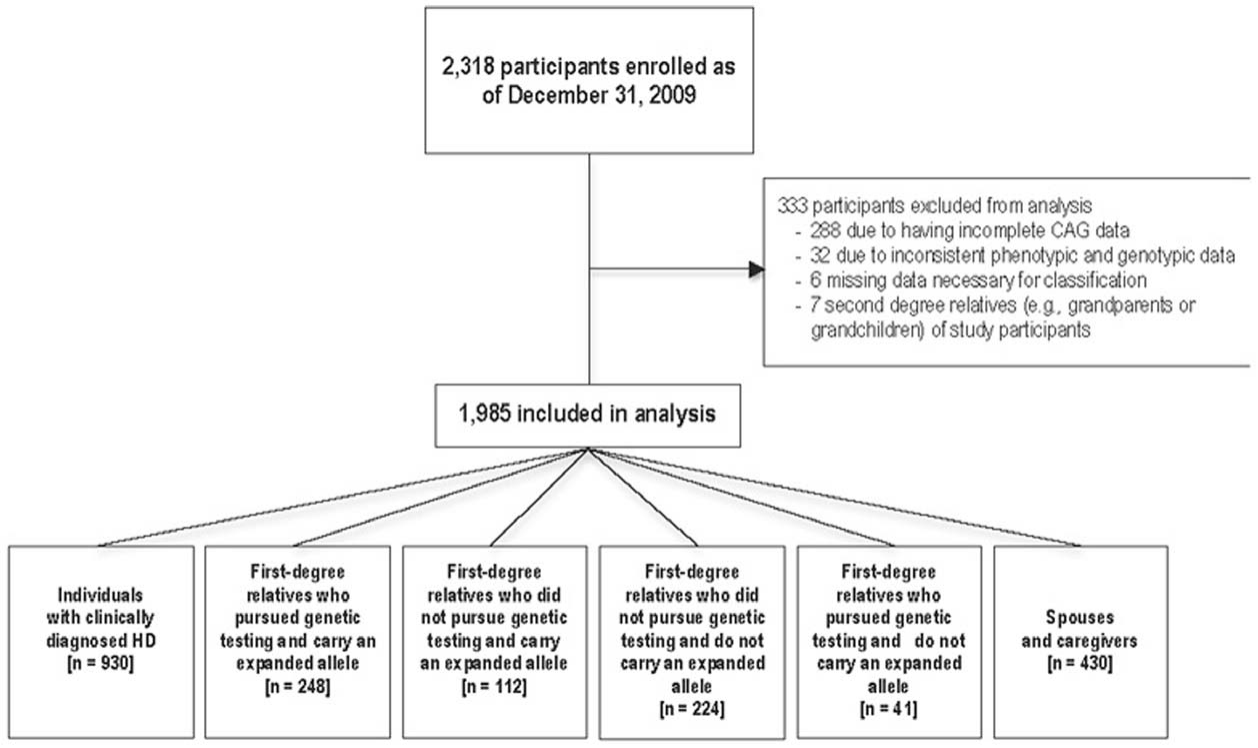
Enrollment and baseline classification of COHORT research participants.

Between February 14, 2006 and December 31, 2009, 2,318 participants enrolled in the COHORT study. Data from 333 (14.4%) participants were excluded from this analysis for the following reasons: 288 had incomplete genotypic data (e.g., absence of CAG repeat length data), 32 had inconsistent genotypic and clinical data (e.g., spouse or caregiver with an expanded CAG allele), seven were second-degree relatives excluded due to low enrollment, and six were missing data necessary for classification of an individual into a group [**Figure 1**]**.**

### Demographic characteristics and medical history

The 1985 participants in this analysis (**Tables 1** **,** **2** **,** **3**) were primarily female (56.3%), had completed at least 12 years of education at the time of enrollment (90.0%), but were not currently employed in the labor force (55.3%).

**Table 1:** Baseline demographics of the COHORT population by group.

	Groups that carry an expanded allele			Groups that do not carry an expanded allele			
	Individuals with clinically diagnosed HD[n = 930]	First-degree relatives who pursued genetic testing and carry an expanded allele[n = 248]	First-degree relatives who did not pursue genetic testing and carry an expanded allele[n = 112]	First-degreerelatives who did not pursue genetic testing and do not carry an expanded allele[n = 224]	First-degree relatives who pursued genetic testing and do not carry an expanded allele[n = 41]	Spousesand caregivers[n = 430]	P-value*
**Group definition**							
(CAG)_n_ of 36 or greater	Yes	Yes	Yes	No	No	No	-
Genetic testing pursued prior to baseline	Some	Yes	No	No	Yes	No	-
Clinically diagnosed with HD	Yes	No	No	No	No	No	-
**Demographics**							
Age [years]	51.9 (12.0)	40.9 (12.5)	40.9 (12.9)	47.2 (14.3)	43.9 (12.2)	52.4 (12.3)	<0.001
Female [%]	51.6	64.1	61.6	69.2	61.0	53.5	<0.001
White [%]	93.2	96.0	90.2	91.0	97.6	94.2	0.11
Ethnicity [% reporting Spanish origin]	3.9	4.4	1.8	4.5	4.9	4.7	0.82
Education [% completing high school or more]	89.4	90.7	88.4	93.7	73.2	90.7	0.004
Employment status [% currently in labor force]	16.8	68.6	72.3	74.4	65.9	66.5	<0.001
Marital status [% currently married]	60.2	62.1	56.3	60.1	61.0	89.5	<0.001

Values are listed as mean (standard deviation) unless otherwise noted.

COHORT = Cooperative Huntington Observational Research Trial; HD = Huntington disease; (CAG)_n_ = cytosine-adenine-guanine repeat length in the *Huntingtin* allele; UHDRS = Unified Huntington's Disease Rating Scale.

*P-values refer to overall tests of heterogeneity across all six groups.

**Table 2:** Baseline clinical characteristics and genetics of the COHORT population by group.

	Groups that carry an expanded allele			Groups that do not carry an expanded allele			
	Individuals with clinically diagnosed HD[n = 930]	First-degree relatives who pursued genetic testing and carry an expanded allele[n = 248]	First-degree relatives who did not pursue genetic testing and carry an expanded allele[n = 112]	First-degreerelatives who did not pursue genetic testing and do not carry an expanded allele[n = 224]	First-degree relatives who pursued genetic testing and do not carry an expanded allele[n = 41]	Spousesand caregivers[n = 430]	P-value*
**Medical history**							
History of at least one suicide attempt [%]	7.1	4.8	3.6	1.8	7.3	1.2	<0.001
**Clinical characteristics** **							
Physical features and vital signs							
Height [centimeters]	170.5 (9.9)	169.0 (9.4)	169.1 (10.3)	168.2 (9.8)	171.6 (9.7)	169.3 (10.3)	0.11
Weight [kilograms]	74.0 (16.8)	76.4 (21.7)	75.3 (17.9)	79.4 (21.7)	80.8 (17.8)	83.6 (21.5)	<0.001
Body mass index [kilograms/meter^2^]	25.4 (5.0)	26.6 (6.7)	26.2 (5.3)	27.9 (6.7)	27.3 (4.9)	29.1 (6.5)	<0.001
Pulse [beats/minute]	74.5 (12.3)	72.7 (11.4)	71.0 (10.3)	69.5 (11.2)	71.4 (10.4)	69.7 (10.5)	<0.001
Systolic blood pressure [millimeters of mercury]	123.6 (17.0)	123.2 (15.3)	123.5 (16.3)	128.3 (17.4)	125.3 (15.4)	129.5 (16.6)	<0.001
Diastolic blood pressure [millimeters of mercury]	76.1 (10.6)	76.6 (10.2)	76.4 (11.6)	77.5 (10.7)	76.6 (10.0)	78.5 (11.8)	0.002
**Genetics**							
(CAG)n of longer allele	44.2 (4.1)	42.6 (2.8)	42.1 (2.4)	20.0 (3.5)	20.5 (4.2)	20.3 (3.6)	<0.001
(CAG)n of shorter allele†	18.5 (3.5)	18.8 (3.5)	18.1 (3.0)	18.4 (2.4)	18.8 (2.6)	18.6 (2.3)	0.43

Values are listed as mean (standard deviation) unless otherwise noted.

COHORT = Cooperative Huntington Observational Research Trial; HD = Huntington disease; (CAG)_n_ = cytosine-adenine-guanine repeat length in the *Huntingtin* allele; UHDRS = Unified Huntington's Disease Rating Scale.

*P-values refer to overall tests of heterogeneity across all six groups.

**For motor and behavioral measures, higher scores reflect greater impairment. For cognitive, independence, and functional measures, higher scores reflect less impairment.

† For participants who do not carry an expanded allele, the (CAG)_n_ of the shorter allele represents the average of two normal alleles.

**Table 3:** Baseline motor, behavioral, and cognitive characteristics of the COHORT population by group.

	Groups that carry an expanded allele			Groups that do not carry an expanded allele			
	Individuals with clinically diagnosed HD[n = 930]	First-degree relatives who pursued genetic testing and carry an expanded allele[n = 248]	First-degree relatives who did not pursue genetic testing and carry an expanded allele[n = 112]	First-degreerelatives who did not pursue genetic testing and do not carry an expanded allele[n = 224]	First-degree relatives who pursued genetic testing and do not carry an expanded allele[n = 41]	Spousesand caregivers[n = 430]	P-value*
**Motor assessment** **							
Total UHDRS motor assessment [0–124]	39.1 (18.3)	6.8 (10.6)	6.9 (11.3)	2.3 (3.3)	1.2 (2.5)	1.5 (2.8)	<0.001
Total maximal chorea score [0–28]	10.1 (5.0)	1.4 (3.2)	2.0 (4.2)	0.2 (0.6)	0.07 (0.4)	0.03 (0.3)	<0.001
Total maximal dystonia score [0–20]	3.5 (3.8)	0.4 (1.5)	0.3 (1.2)	0.03 (0.2)	0 (0)	0.02 (0.1)	<0.001
**Behavioral assessment**							
UHDRS Behavioral frequency [0–44]	7.0 (6.1)	6.9 (6.9)	6.4 (6.2)	4.5 (4.5)	4.9 (4.7)	4.4 (4.2)	<0.001
UHDRS Behavioral frequency×severity [0–176]	14.0 (15.4)	12.7 (17.7)	11.2 (15.2)	6.5 (9.1)	7.8 (12.5)	6.2 (9.0)	<0.001
**Cognitive assessment**							
Mini Mental State Examination [0–30]	25.0 (4.6)	28.6 (1.9)	28.2 (2.5)	29.0 (1.6)	29.4 (1.3)	29.1 (1.5)	<0.001
UHDRS Verbal fluency	21.9 (12.5)	38.1 (14.1)	36.6 (14.9)	39.8 (11.7)	42.2 (10.2)	40.1 (12.1)	<0.001
UHDRS Symbol digit modalities test	23.6 (11.2)	45.4 (12.5)	45.2 (12.4)	49.5 (11.8)	49.2 (10.4)	45.9 (10.2)	<0.001
UHDRS Stroop color naming	44.3 (16.9)	70.9 (17.8)	71.1 (16.0)	76.9 (15.8)	72.8 (14.5)	73.3 (14.8)	<0.001
UHDRS Stroop word reading	58.0 (21.9)	90.1 (21.5)	90.4 (19.2)	95.8 (16.3)	99.2 (17.8)	94.6 (19.3)	<0.001
UHDRS Stroop interference	25.5 (12.5)	42.8 (12.9)	41.2 (12.3)	44.8 (12.0)	50.6 (23.7)	41.8 (12.1)	<0.001
UHDRS Independence assessment	79.1 (16.2)	97.2 (7.2)	99.1 (3.9)	99.9 (0.9)	99.5 (3.1)	99.8 (1.4)	<0.001
UHDRS Functional assessment	18.7 (6.0)	24.4 (1.9)	24.8 (1.1)	24.9 (0.3)	24.9 (0.5)	24.9 (1.3)	<0.001
UHDRS Total functional capacity	8.1 (3.4)	12.3 (1.7)	12.7 (1.0)	12.9 (1.0)	12.9 (0.5)	12.9 (0.6)	<0.001

Values are listed as mean (standard deviation) unless otherwise noted.

COHORT = Cooperative Huntington Observational Research Trial; HD = Huntington disease; (CAG)_n_ = cytosine-adenine-guanine repeat length in the *Huntingtin* allele; UHDRS = Unified Huntington's Disease Rating Scale.

*P-values refer to overall tests of heterogeneity across all six groups.

**For motor and behavioral measures, higher scores reflect greater impairment. For cognitive, independence, and functional measures, higher scores reflect less impairment.

At baseline, 94 (4.7%) of the 1,985 participants reported at least one prior suicide attempt. Individuals with clinically diagnosed HD were more likely to have attempted suicide (7.1%) than caregivers or spouses (1.2%; p<0.001) and all other study participants (2.7%; p<0.001). The most commonly used medications among those with clinically diagnosed HD were anti-depressants (32.4%), multivitamins (27.4%) and anti-psychotics (24.5%) and for all other groups were multivitamins (27.4%), lipid modifying agents (18.9%), and anti-depressants (11.5%) (**Table 4**).

**Table 4:** Three most common medication classes used by participants in the COHORT study.

	Manifest HDn = 930	n(%)	Pre-manifest HDn = 248	n(%)	At-risk and carrying an expanded allelen = 112	n(%)	At-risk but not carrying an expanded allelen = 224	n(%)	First degree relatives known not to carry an expanded allelen = 41	n(%)	Control participants (spouses and caregivers)n = 430	n(%)
**1**	Antidepressants	301(32)	Multivitamins	65(26)	Multivitamins	27(24)	Multivitamins	55(25)	Multivitamins	7(17)	Multivitamins	135(31)
**2**	Multivitamins	255(27)	Lipid-modifying agents	51(21)	Antidepressants	13(12)	Lipid-modifying agents	39(17)	Calcium;Lipid-modifying agents	5(12)	Lipid-modifying agents	94(22)
**3**	Antipsychotics	228(25)	Antidepressants	48(19)	Lipid-modifying agents	10(9)	Antiinflammatory and antirheumatic products;Non-steroids	26(12)	Combinations;Unspecified herbals	4(10)	Combinations*	60(14)

*Combinations = products containing two or more active ingredients.

### Clinical characteristics

Weight and body mass index varied significantly across groups. Individuals with clinically diagnosed HD weighed less (74.0 kg) than spouses and caregivers (83.6 kg; p<0.001) and had a lower body mass index (25.4 kg/m^2^ vs. 29.1 kg/m^2^; p<0.001). First-degree relatives who carried an expanded allele but were not clinically diagnosed with HD weighed less (76.0 kg vs. 79.6 kg; p = 0.01) and tended to have a lower body mass index (26.5 kg/m^2^ vs. 27.8 kg/m^2^; p = 0.06) than first-degree relatives without an expanded allele.

Motor, behavioral, cognitive, and functional scores on the UHDRS differed across groups, as those with clinically diagnosed HD had worse scores than all other participants (p<0.001 for all aspects of the UHDRS). Similarly, MMSE scores differed significantly across groups, and those with clinically diagnosed HD had worse scores (25.0) than spouses and caregivers (29.1; P<0.001). First-degree relatives with expanded alleles who were not clinically diagnosed with HD had lower MMSE scores (28.5) than all first-degree relatives without an expanded allele (29.1; p = 0.008).

### Distribution of CAG repeat lengths

**Table 5** and **6** show the distribution of participants' CAG repeat lengths for the larger and shorter *Huntingtin* alleles. Fifty individuals (2.5%) had a repeat length on their larger allele in the high normal range, and none had clinically diagnosed HD. Fifty-three individuals (2.7%) had CAG repeat lengths on their larger allele associated with reduced penetrance. Of these, 15 (28.3%) were diagnosed with HD prior to their baseline visit.

**Table 5:** CAG repeat length of the larger *Huntingtin* allele in different groups in the COHORT study.

	Groups that carry an expanded allele			Groups that do not carry an expanded allele		
	Individuals with clinically diagnosed HD[n = 930]	First-degree relatives who pursued genetic testing and carry an expanded allele[n = 248]	First-degree relatives who did not pursue genetic testing and carry an expanded allele[n = 112]	First-degreerelatives who did not pursue genetic testing and do not carry an expanded allele[n = 224]	First-degree relatives who pursued genetic testing and do not carry an expanded allele[n = 41]	Spousesand caregivers[n = 430]
**(CAG)_n_ of 26 or less [n (%)]**	0	0	0	209 (93.3)	38 (92.7)	398 (92.6)
**(CAG)_n_ between 27 and 35 (inclusive) [n (%)]**	0	0	0	15 (6.7)	3 (7.3)	32 (7.4)
**(CAG)_n_ between 36 and 39 (inclusive) [n (%)]**	15 (1.6)	25 (10.1)	13 (11.6)	0	0	0
**(CAG)_n_ of 40 or more [n (%)]**	915 (98.4)	223 (89.9)	99 (88.4)	0	0	0

**Table 6:** CAG repeat length of the shorter *Huntingtin* allele in different groups in the COHORT study.

	Groups that carry an expanded allele			Groups that do not carry an expanded allele		
	Individuals with clinically diagnosed HD[n = 930]	First-degree relatives who pursued genetic testing and carry an expanded allele[n = 248]	First-degree relatives who did not pursue genetic testing and carry an expanded allele[n = 112]	First-degreerelatives who did not pursue genetic testing and do not carry an expandedallele[n = 224]	First-degree relatives who pursued genetic testing and do not carry an expanded allele[n = 41]	Spousesand caregivers[n = 430]
**(CAG)_n_ of 26 or less [n (%)]**	893 (96.0)	236 (95.2)	110 (98.2)	224 (100)	41 (100)	430 (100)
**(CAG)_n_ between 27 and 35 (inclusive) [n (%)]**	36 (3.9)	12 (4.8)	2 (1.8)	0	0	0
**(CAG)_n_ between 36 and 39 (inclusive) [n (%)]**	1 (0.1)	0	0	0	0	0
**(CAG)_n_ of 40 or more [n (%)]**	0	0	0	0	0	0

COHORT = Cooperative Huntington Observational Research Trial; HD = Huntington disease; (CAG)_n_ = cytosine-adenine-guanine trinucleotide repeat length.

**Figure 2:**
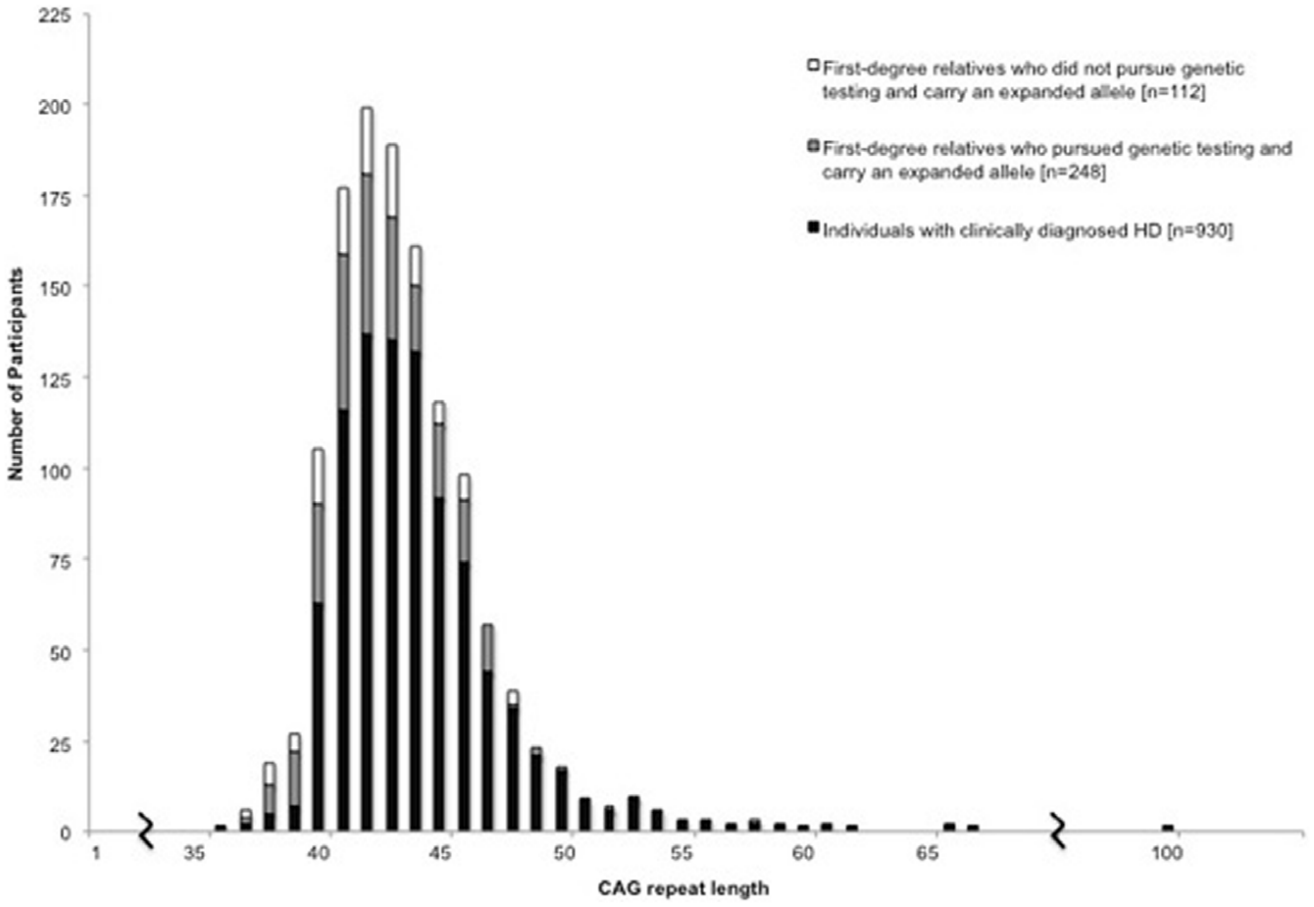
Distribution of the CAG repeat length of the larger *Huntingtin* allele for all individuals carrying an expanded allele in the COHORT study.

The average CAG repeat length of the larger allele was 44.2±4.1 for individuals with clinically diagnosed HD (range 36 to 100 repeats) and 42.6±2.8 for first-degree relatives with an expanded allele (range 38 to 58 repeats) [**Figure 2**].

### Reportable events

Through December 31, 2009, one completed suicide in an individual with clinically diagnosed HD and eleven suicide attempts (nine in individuals with clinically diagnosed HD) occurred (**Table 7**). The individual who committed suicide had reported a prior history of depression and multiple previous suicide attempts. For the eleven participants who attempted suicide, nine (82%) were female, the mean age was 43.4 (range 26–55), seven (64%) had reported a prior history of depression, and four (36%) had reported a history of at least one previous suicide attempt. For those with clinically diagnosed HD, the most commonly reported cause was disease progression or complications (n = 8), followed by cardiac etiology (n = 5) and respiratory etiology (n = 5). The main reasons for premature withdrawal were voluntary withdrawal of consent (n = 20), lost to follow-up (n = 11), and caregiver decision (n = 5).

**Table 7:** Reportable events within the COHORT study by group, 2006–2009.

	Groups that carry an expanded allele			Groups that do not carry an expanded allele		
	Individuals with clinically diagnosed HD[n = 930]	First-degree relatives who pursued genetic testing and carry an expanded allele[n = 248]	First-degree relatives who did not pursue genetic testing and carry an expanded allele[n = 112]	First-degreerelatives who did not pursue genetic testing and do not carry an expanded allele[n = 224]	First-degree relatives who pursued genetic testing and do not carry an expanded allele[n = 41]	Spousesand caregivers[n = 430]
**Suicide [n (%)]**	1 (0.1)	0	0	0	0	0
**Suicide attempt [n (%)]**	9 (1.0)	2 (0.8)	0	0	0	0
**Deaths other than suicide [n (%)]**	18 (1.9)	1 (0.4)	0	0	0	0
**Premature withdrawal [n (%)]**	29 (3.1)	4 (1.6)	0	4 (1.8)	0	13 (3.0)

COHORT = Cooperative Huntington Observational Research Trial; HD = Huntington disease.

### Optional assessments

Participation in the optional assessments was high, as 97% of participants consented to provide specimens for lymphoblastoid cell lines, 98% consented to be contacted for future studies, and 70% completed the Family History Questionnaire.

## Discussion

In a large, multi-national observational study of individuals from families affected by HD, the groups enrolled differed in their demographic, clinical, and genetic features at baseline. While many differences observed were expected, several are noteworthy.

Consistent with the growing evidence that changes occur in individuals who carry an expanded allele prior to the clinical (motor) diagnosis of HD [16], these individuals had worse cognitive performance on the UHDRS and MMSE and weighed less than those without expanded alleles. A recent report found that nearly 40% of individuals who knew they carried an expanded *Huntingtin* allele but were not diagnosed with HD met criteria for mild cognitive impairment [17]. This study also adds evidence that weight loss may precede the clinical onset of symptoms [18] and is consistent with HD transgenic mice studies showing that weight loss precedes motor symptoms [19]–[20]. Future longitudinal assessment will more fully characterize the prodrome of HD.

The results also provide guidance on suicide risks among individuals from families with HD. Among individuals with HD, suicide is more common than in the general population [16], [21]–[24] and accounts for 5 to 7% of deaths [22]–[23], [25]. Over 25% of individuals with HD attempt suicide at least once [22]. Through 2009 only one suicide occurred in COHORT, and among 930 individuals with clinically diagnosed HD followed for more than 2000 participant-years, only nine suicide attempts occurred. The rate among individuals in other groups is lower. While the low rate may be due to low ascertainment, suicides and suicide attempts are prospectively assessed and reported within three working days after a site becomes aware of the event. The study's prospective annual assessment of mental health and the requirement for referral to a mental health professional when pre-specified mood and ideation thresholds are met may be contributing to the relatively low rates observed. Another factor is that COHORT's study population is not a random sample of the general HD population. However, it is likely representative of clinical trial participants and thus can serve as a useful comparator for investigations of experimental therapeutics.

Because of its large size, COHORT also provides an excellent opportunity to examine the prevalence of individuals who have CAG repeat lengths in the high normal range and in the range associated with reduced penetrance. In the present study population, 50 individuals had a larger allele in the high normal range and an additional 50 had a shorter allele in that range. Of these, 17 (5.1%) were among the 336 first-degree relatives who had not pursued prior genetic testing, and 15 (5.2%) were among the 289 first-degree relatives who had pursued genetic testing prior to the onset of HD. A previous study reported that 7% of individuals pursuing genetic testing for HD had CAG repeat lengths in the high normal range [26]. Together these results suggest that the prevalence of high normal alleles among individuals at-risk for HD is not rare. Based on modeling estimates, the likelihood that a male high normal allele carrier will have offspring with an expanded penetrant allele is small, on the order of one in a thousand [6], [27]. Although the current COHORT sample is not sufficiently large to test this estimate, future data linking across generations using the Family History Questionnaire could better define the stability of repeat lengths between generations, and longitudinal follow-up will help to characterize the clinical evolution of these individuals.

COHORT also has 53 individuals with CAG repeat lengths associated with reduced penetrance on their larger allele. Twenty-five (8.7%) of the individuals without clinically diagnosed HD who had pursued pre-symptomatic testing and 13 (3.9%) of first-degree relatives who had not pursued genetic testing had repeat lengths associated with reduced penetrance. These results correspond to a recent report that 5% of individuals undergoing pre-symptomatic testing have repeat lengths associated with reduced penetrance [26]. In COHORT, 15 (28.3%) of these 53 individuals had received a clinical diagnosis of HD prior to enrollment in the study. Current estimates suggest that at least 40% of individuals with a repeat in this range will be asymptomatic at age 65 [4], which can be verified through prospective assessments in COHORT.

Beyond this report, COHORT's value lies in its potential to serve as an open resource for HD investigators and to inform the design and conduct of future clinical trials. The phenotypic and genotypic data derived and the current biological specimens can be accessed by researchers anywhere through a brief proposal process by contacting cohort.projectmanager@ctcc.rochester.edu. Additional genetic evaluations of the influence of non-expanded *Huntingtin* CAG repeats, the *GRIK2* gene [28], and other potential genetic modifiers of HD pathogenesis are underway. Many clinically-oriented questions remain, including a detailed longitudinal history of the cardinal features of HD; factors that influence those features; the long-term safety of approved and experimental therapies for HD; and trends in the clinical care of those with and at-risk for HD.

In addition to answering important research questions, COHORT can inform and enhance the investigation of experimental therapeutics. The selection of outcome measures that are sensitive to changes in different features of the disease with low variability is an important decision for clinical trials. In addition, COHORT can be used to determine the relative number of research participants available based on different inclusion criteria for clinical trials. Finally, the study can and has been used to identify potential research participants and sites for clinical trials based on key entry criteria. For example, in a study looking at a treatment for cognitive impairment in HD, COHORT could be used to identify research participants with a MMSE score below a specific threshold.

While COHORT has tremendous value and potential, it has several limitations. The study population – overwhelmingly white, relatively highly educated, and currently centered in three countries – may not be representative of the broader HD population. Study participants were enrolled primarily at academic research centers, which might limit the generalizability to individuals lacking access to these clinics, including those residing in nursing facilities. A sister European study called REGISTRY can address some of these limitations [29]. Another limitation is that COHORT currently has few biological markers tied to the study that can take advantage of the large and valuable clinical dataset. Additional studies could be incorporated into COHORT to allow better coupling of phenotypic data with the growing knowledge of biomarkers in HD [30]. Like other large, multi-center studies, ensuring the completeness of the clinical and genetic data captured in the study can be difficult. For this report, we excluded data from approximately 15% of research participants principally due to incomplete genetic data or potentially inconsistent clinical and genetic data, which is currently under investigation. Future collection and verification of the data over time will allow for more complete reporting.

This report details the baseline characteristics of nearly 2000 individuals from families affected by HD, demonstrates clinical differences and their size, and highlights over 150 individuals with high normal or reduced penetrant *Huntingtin* alleles. More importantly, this report establishes the foundation for valuable longitudinal analyses of this population, a resource for HD investigators globally, and a powerful tool for designing and conducting future trials of experimental therapeutics for HD.

## Supporting Information

Protocol S1 Trial Protocol.
(DOC)
