# Correction: Characterization of a Large Group of Individuals with Huntington Disease and Their Relatives Enrolled in the COHORT Study

**DOI:** 10.1371/annotation/25881bc7-922d-4472-9efd-f0896b1a3499

**Published:** 2012-08-30

**Authors:** E. Ray Dorsey, the Huntington Study Group COHORT Investigators

There was an error in the author byline. The correct byline should read: the Huntington Study Group COHORT Investigators. Author E. Ray Dorsey remains corresponding author of the article. The correct citation is: the Huntington Study Group COHORT Investigators (2012) Characterization of a Large Group of Individuals with Huntington Disease and Their Relatives Enrolled in the COHORT Study. PLoS ONE 7(2): e29522. doi:10.1371/journal.pone.0029522

