# Supplementary material for: Characterization of a Large Group of Individuals with Huntington Disease and Their Relatives Enrolled in the COHORT Study
Source: PLoS One. 2012 Feb 16;7(2):e29522. doi: 10.1371/journal.pone.0029522 (PMC3281013; doi:10.1371/journal.pone.0029522)
Supplement: Protocol S1 — Trial Protocol. (DOC) [file pone.0029522.s001.doc]

##### PROTOCOL

##### Version Date – August 31, 2006

##### Amendment #1

Title: **Co**operative **H**untington’s Disease **O**bservational **R**esearch **T**rial **(COHORT)**

###### Sponsored by: HP Therapeutics Foundation, Inc

###### Huntington Study Group

Study Principal Investigator: Ira Shoulson, MD

Study Co-Principal Investigators: Tatiana Foroud, PhD

James Gusella, PhD

**INVESTIGATOR'S AGREEMENT**

By signing below, I confirm that I have read this protocol and agree that it contains all necessary details for conducting this study. I will conduct the study according to the procedures described in this protocol.

Site Investigator's Name

Site Investigator's Address

(Type or Print)

Site Investigator's Signature Date

**COHORT PROTOCOL SYNOPSIS**

| **Protocol Title** | **Co**operative **H**untington’s **O**bservational **R**esearch **T**rial **(COHORT)** |
| --- | --- |
| **Type of Study** | Observational Study without experimental treatment |
| **Planning Committee** | R. Blumenstein, M. Conneally, T. Foroud, J. Gray, J. Gusella, M. Harrison, E. Kayson, K. Kieburtz, M. Nance, D. Oakes, K. O’Donoghue, M. Romer, C. Ross, A. Shinaman, I. Shoulson, O. Suchowersky, T. Tempkin, A. Tobin |
| **Steering Committee** | Ira Shoulson (PI), Tatiana Foroud (co-PI), James Gusella (co-PI), Michael Conneally, Madaline Harrison, Richard Myers, Martha Nance, David Oakes, Julie Stout, Oksana Suchowersky, Terry Tempkin, Robi Blumenstein *(ex-officio)*, Jacqueline Gray *(ex-officio)*, Elise Kayson *(ex-officio)*, Karl Kieburtz *(ex-officio)*, Kelley O’Donoghue *(ex-officio)*, Aileen Shinaman *(ex-officio)* |
| **Study Centers** | Approximately 41 HSG sites in the United States, Canada, and Australia |
| **Study Period** | Subjects will be asked to participate in as many yearly study visits as possible. |
| **Study Objective** | To collect prospective data from individuals who are part of an HD family, in order to relate phenotypes between individuals and families with each other and genetic factors in order to learn more about HD, develop potential treatments for HD, and to plan for future research studies of experimental drugs aimed at slowing or postponing the onset and progression of HD. |
| **Study Population** | Subjects will either have the signs and symptoms of Huntington’s disease (HD) or be a member of an HD family. |
| **Number of Subjects** | This study will include as many subjects who are eligible and willing to participate. We expect that thousands of individuals will participate. |
| **Inclusion Criteria** | For those 18 years of age and older, there are four different types of individuals who may be eligible to participate in COHORT   1. An individual with clinically diagnosed features of HD in the setting of a confirmatory family history or an individual known to carry the HD gene as confirmed by DNA testing (individual is defined as the ‘proband’). 2. A first-degree relative (parents, siblings, or children) of an individual with clinically diagnosed features of HD in the setting of a confirmatory family history or of an individual known to carry the HD gene as confirmed by DNA testing. 3. A grandparent or grandchild of an individual participating in COHORT who meets criterion 1. 4. A HD family member who has no risk for HD due to no family history (spouses of proband only) or negative gene testing. This individual must have an affected or gene positive spouse or family member participating in COHORT to be included.   For individuals under the age of 18, only those with clinically diagnosed features of HD in the setting of a confirmatory family history or clinically diagnosed features of HD with a positive gene test will be eligible to participate in COHORT (individual is defined as the ‘proband’).  A confirmatory family history of HD is defined by the presence of either: 1) a parent or sibling with clinically diagnosed features of HD or 2) a first-degree relative (parent, sibling, child) known to be a carrier of the HD gene through pre-symptomatic DNA testing.  Subjects may be male or female. All subjects must be able to provide consent for themselves, have a parent/guardian who can provide parental permission, or have an authorized representative who can provide consent. |
| **Exclusion Criteria** | Anyone who does not fit the criteria stated under “Inclusion Criteria.” |
| **Study Design** | At the baseline study visit, study personnel will administer the Unified Huntington’s Disease Rating Scale 99 (UHDRS 99), the Mini-Mental State Examination (MMSE), and a complete physical and neurological examination will be conducted. Study personnel will collect information about medical history and concomitant medications. All subjects 18 years of age and older, will have a single blood specimen collected for determination of genetic differences related to Huntington’s disease. DNA derived from the blood sample will be used to obtain the subject’s genotype for the CAG repeat in the HD gene, which is associated with susceptibility to the disorder, and to obtain genotypes for authenticated polymorphisms in any other genes previously shown to modify timing or expression of disease features.    At the baseline visit, subjects will be asked to complete the following **optional** study procedures.   1. Family History Questionnaire (FHQ) – Any subject 18 years of age and older with a family history of Huntington’s disease will be asked to participate. The FHQ will only collect information about the side of the family affected by Huntington’s disease. The FHQ will collect information about the spouse, parents, grandparents, children, siblings, aunts, uncles, nieces, and nephews. The questionnaire will collect name, sex, birth date, and date of death, if applicable. For those affected with HD, it will also collect information regarding age of HD diagnosis and if a physician diagnosed the HD. The subject will be given the option of completing the questionnaire during the study visit or can take the questionnaire home with a stamped, addressed envelope to complete and mail back to the study site. If the completed questionnaire is not received within two months of the study visit, the site will follow-up with the subject. 2. Storage of Biological Specimens –All subjects 18 years of age and older will be given the **option** of allowing blood and urine samples to be collected for storage in a specimen repository for future research in Huntington’s disease. If the subject agrees to participate in this aspect of the study, blood will be drawn at every visit and urine at specified visits.   At each yearly visit, the following will take place:   1. Unified Huntington’s Disease Rating Scale 99 (UHDRS 99) 2. Mini-Mental State Examination (MMSE) 3. A brief medical evaluation will be conducted, including the measurement of height, weight and vital signs. 4. Information collected about medical and mental health events, which have occurred since the last visit and changes in concomitant medications. 5. Subjects will be given the opportunity to reevaluate their decision regarding participation in the optional procedures. 6. Family History Questionnaire - If the subject agreed to participate in the family history questionnaire, he or she will be asked to provide information about new deaths and onsets of HD. 7. Collection of Biological Specimens – If the subject agreed to have blood stored in the repository, a blood sample will be collected. If the subject agreed to have urine stored in the repository, urine will be collected at either the 2^nd^, 3^rd^, or 4^th^ yearly visit.   *Note: Neither subjects nor investigators will ever receive individual results from procedures performed in this study. The DNA genotyping for the HD CAGn and genotyping for authenticated polymorphisms in other genes previously shown to modify timing or expression of disease features will be performed in a research lab and therefore the results are experimental data. Under no circumstances will the results be reported to the sites or to the subjects. If a subject decides to seek DNA genotyping for the HD CAGn for the purposes of genetic testing while participating in this study, he/she may do so through the available HD predictive testing centers.*  Study personnel will work in conjunction with the subject to create a mechanism to best contact the subject to set up yearly study visits. This will be used to maximize subject retention in the study. Subjects will be given the **option** of allowing study personnel to contact them in-between visits to collect additional information or to provide updates regarding the study. |
| **Phenotypic Measure(s)** | **Unified Huntington’s Disease Rating Scale 99** – to obtain information about disease progression over four major domains: motor function, cognition, behavior, and functional capacity.  **Mini-Mental State Examination** - to obtain a brief measure of cognitive status in adults. |
| Genotypic Measure(s) | **Family History Questionnaire** – to obtain valid correlations of the genotype and phenotype it is critical that documentation of disease variation is collected. Disease variation is collected through the family history questionnaire.  **CAG repeat length/Other genetic polymorphisms** - to obtain an accurate measure of the expanded CAG repeat within the coding region of the novel 4p16.3 *HD* gene. This will allow for the comparison of genotypic information against phenotypic information to help define the mechanism of Huntington’s disease and factors that affect its onset, progression, and disease manifestations. Genotypes at modifier loci may be obtained to obtain accurate measures of the subject’s genotype for authenticated genes that have previously shown to modify the timing or presentation of disease features. This will allow for the comparison of genotypic information against phenotypic information, to help define the mechanisms involved in Huntington’s disease pathogenesis and factors that affect its onset, progression, and disease manifestations. |
| **Sample Size Considerations** | In performing an observational cohort study, the larger the sample size used for data analysis, the more applicable the results are to the population as a whole. Therefore, COHORT will allow each site to enroll as many subjects as are eligible and willing to participate. Several genetic studies are proposed to delineate the genetic and environmental effects on relevant phenotypic events such as phenoconversion or development of signs of HD. Initial analyses will estimate the proportion of phenotypic variation due to genetic factors and will be followed by studies designed to identify the DNA sequence changes that underlie the phenotypic variation. To estimate the influence of genetic factors, typically samples of 500-1,000 sibling pairs are required to detect genetic contributions of as little as 20%. This would typically be considered the lower bound for a covariate that might be identified, which could act as a meaningful covariate for therapeutic trials aimed at delaying the phenotypic event (e.g., HD phenoconversion). To identify the genes and sequence variation underlying the trait, typically even larger samples are required, but they can include various types of related individuals such as siblings, half siblings, parent-offspring, cousins, etc. A sufficiently large sample size of a relevant phenotypic event (e.g., phenoconversion or development of signs of HD) will provide adequate statistical power to detect a relevant risk factor (e.g., genetic polymorphism or environmental effect) that influences the phenotypic event and that might meaningfully serve as a covariate for therapeutic trials aimed at delaying the phenotypic event (e.g., HD phenoconversion). |

Cooperative Huntington’s Disease Observational Research Trial **(COHORT)**

**Table of Contents**

1. Study Objective
2. Background and Rationale
3. Discussion of Study Design
4. Characteristics of the Research Population
   1. Number of Subjects
   2. Gender of Subjects
   3. Age of Subjects
   4. Racial and Ethnic Origin
   5. Inclusion Criteria
   6. Exclusion Criteria
   7. Vulnerable Subjects/Subject Capacity
5. Subject Identification, Recruitment, and Consent/Assent
   1. Method of Subject Identification and Recruitment
   2. Process of Consent
   3. Documentation of Consent/Assent
   4. Consent/Assent form
   5. Waiver of Consent/Waiver of Authorization
6. Study Procedures
   1. Methods and Procedures
   2. Procedure Descriptions
      1. Unified Huntington’s Disease Rating Scale 99
      2. Mini-Mental State Examination
      3. CAG Genotyping/Other Genetic Polymorphisms
      4. Contact Between Study visits
      5. Family History Questionnaire (FHQ)
      6. Specimen Repository
      7. Future Contact Regarding Research Studies
   3. Costs to the Subject
   4. Payment
   5. Data Management, Data Storage and Confidentiality
7. Risk/Benefit Assessment
   1. Potential Risks
   2. Protection Against Risks
   3. Potential Benefit
   4. Alternatives to Participation
   5. Withdrawal from Participation
8. Statistical Considerations
   1. Sample Size Calculation
   2. Data Analysis
9. Consultants
   1. Planning Committee
   2. Steering Committee
10. References

**Cooperative Huntington’s Disease Observational Research Trial (COHORT)**

# STUDY OBJECTIVE

##### COHORT (Cooperative Huntington Observational Research Trial) is a coordinated research effort by Huntington Study Group research centers worldwide to prospectively collect data from consenting individuals who are affected by Huntington’s disease (HD) and who are part of an HD family. The systematically accrued data from annual prospective assessments will relate clinical characteristics (phenotypes) between families with genetic and environmental factors. The knowledge from these relationships will better inform us about the onset and progression of HD, help identify potential interventions for HD, and aid in planning research studies of experimental treatments aimed at slowing or postponing the onset of HD. The consented collection of biological samples will further provide research material and correlative data for scientists to identify biomarkers that parallel the development and progression of HD. Identification of biomarkers will in turn contribute to our understanding of HD and enhance the efficiency and power of disease-modifying therapeutic trials.

# BACKGROUND AND RATIONALE

Huntington’s Disease (HD) is a delayed-onset autosomal dominant neurodegenerative disorder affecting approximately 30,000 people in the United States.[[1]](#endnote-2),[[2]](#endnote-3) Another 60,000 individuals carry the HD gene but remain yet unaffected. HD is caused by an unstable expansion of the cytosine-adenine-guanine (CAG) trinucleotide repeat in exon-1 of the IT-15 gene on chromosome 4.[[3]](#endnote-4),[[4]](#endnote-5) Individuals who inherit the mutant gene develop selective neuronal degeneration and eventually motor, cognitive and behavioral abnormalities that cause progressive loss of functional capacity and shortened life.[[5]](#endnote-6), [[6]](#endnote-7),[[7]](#endnote-8),[[8]](#endnote-9),[[9]](#endnote-10),[[10]](#endnote-11),[[11]](#endnote-12) There is no treatment to delay the onset or forestall the progression of illness.[[12]](#endnote-13),[[13]](#endnote-14),[[14]](#endnote-15),[[15]](#endnote-16),[[16]](#endnote-17),[[17]](#endnote-18)

Since the gene mutation responsible for HD was identified in 1991, considerable progress has been made in understanding the etiology and pathogenesis of this disorder. Likewise, incremental gains in clinical research have been achieved involving observational cohort studies of HD patients (UHDRS cohort) and individuals at high risk for having inherited the HD gene (PHAROS) or known carriers of the HD gene (PREDICT-HD). However, more optimal integration of basic and translational HD research with systematically collected human data and biological material would better inform the planning and conduct of clinical research by identifying promising experimental treatments, the factors that influence the onset and progression of illness, and state biomarkers that parallel the onset and course of HD.

The **Co**operative **H**untington **O**bservational **R**esearch **T**rial (**COHORT**) is an ambitious research undertaking aimed at integrating prospective and systematically collected clinical research data (demography, clinical features, family history, genetic characteristics) with access to biological specimens (e.g., blood, urine) obtained from individuals with manifest HD, unaffected individuals known to carry the HD gene, unaffected individuals who are at risk of carrying the HD gene, and family members who have no risk for HD due to no family history or negative HD gene testing (control subjects). Biological specimens and correlative clinical, hereditary and genetic data may be provided (in combination or separately) to qualified scientists who have institutional review board (IRB) reviewed research protocols that aim to advance scientific knowledge, which in turn will lead to treatments that forestall onset and slow the progression of HD.

COHORT was conceived as a wide-based and long-term project to maximize the efficiencies of preclinical research and participation in clinical research (see NIH Roadmap for clinical re-engineering) and at the same time ensuring the privacy and protections for consenting research subjects. COHORT builds on the strong collaborative relationships that have been nurtured among basic scientists (e.g., Cure HD Initiative, Coalition for the Cure), clinical investigators (e.g., Huntington Study Group) and the broad involvement of the HD community (Huntington Project), in North America and worldwide. The increasing relations among these existing collaborative efforts will further enhance prospects for the safe and efficient conduct of COHORT and the incremental knowledge required to advance the experimental therapeutics of HD.

COHORT is the centerpiece of the Huntington Project (<http://www.huntingtonproject.org/>), a worldwide collaborative undertaking to develop treatments that make a difference for HD. The Huntington Project and COHORT are sponsored by HP Therapeutics Foundation, Inc., a not-for-profit corporation that supports a variety of research projects seeking to find treatments for Huntington's disease. COHORT was planned and is overseen by the Huntington Study Group (HSG), (<http://www.huntington-study-group.org/>), a not-for-profit consortium of researchers established in 1993 to improve treatments for HD. The HSG now comprises more than 70 sites worldwide and has carried out 10 multi-center observational and interventional studies involving more than 10,000 individuals affected by HD. COHORT builds on the accrued HSG databases from these studies and its established network of investigators and research subjects.

HD is a uniformly progressive neurodegenerative disorder characterized clinically by movement disorder (typically chorea), behavioral disturbances, and cognitive impairment. The mean age of HD onset is approximately 39 years, after which illness progresses steadily over a period of 15-25 years. Approximately 30,000 people in the United States have clinical manifestations of HD, and an additional 150,000 unaffected individuals (about 60,000 HD gene carriers and 90,000 non-HD gene carriers) are immediately at risk to develop HD. In the United States, only about 5-10% of individuals at immediate risk for HD have chosen to undergo pre-symptomatic DNA testing to learn if they carry the HD gene.

The steady worsening of the motor, cognitive, and behavioral features of HD results in progressive functional decline. Clinical rating scales that chart the progression of illness have been widely used to establish the rate of functional decline in a variety of HD populations.[[18]](#endnote-19),[[19]](#endnote-20),[[20]](#endnote-21),[[21]](#endnote-22),[[22]](#endnote-23),[[23]](#endnote-24) The extent of functional disability correlates well with the extent of basal ganglia degeneration detected by neuroimaging and postmortem examination.Error: Reference source not found,[[24]](#endnote-25),[[25]](#endnote-26),[[26]](#endnote-27),[[27]](#endnote-28)

The **Unified Huntington's Disease Rating Scale** (UHDRS) was developed by the HSG in 1993 and revised in 1999 as UHDRS 99.[[28]](#endnote-29),[[29]](#endnote-30),[[30]](#endnote-31) The UHDRS assesses four major clinical domains of impairment: (1) motor, (2) cognitive, (3) behavioral, and (4) functional capacity. In devising this scale, items were selected that were likely to be sensitive to the early progression of illness. The UHDRS 99, which will be employed in COHORT, has been used at more than 70 HSG sites in North America, Europe, and Australia. The UHDRS has undergone extensive testing of reliability and internal consistency.Error: Reference source not found,Error: Reference source not found,Error: Reference source not found In a study involving three independent clinicians who evaluated the motor performance of 24 HD subjects, the inter-rater reliability was 0.94 (interclass correlation) for the total motor score. The motor and cognitive sections of the UHDRS correlated strongly and significantly with the functional component of the UHDRS. Internal consistency, as measured by Cronbach's alpha was 0.95 for the motor component, 0.90 for the cognitive tests, 0.83 for the behavioral component, and 0.95 for the functional component of the UHDRS.[[31]](#endnote-32) The UHDRS has been used widely in HD clinical trials.[[32]](#endnote-33),[[33]](#endnote-34),[[34]](#endnote-35)

The motor section of the UHDRS assesses motor features of HD using standardized ratings of oculomotor function, dysarthria, chorea, dystonia, gait, and postural stability. The total motor impairment score is the sum of the individual motor ratings; higher scores indicate more severe motor impairment. Cognition is assessed by a phonetic verbal fluency test,[[35]](#endnote-36) Symbol Digit Modality Test,[[36]](#endnote-37) and Stroop Interference Test.[[37]](#endnote-38) Higher scores indicate better cognitive performance. The behavioral assessment measures frequency and severity of symptoms related to altered affect, thought content and coping styles.[[38]](#endnote-39) The total behavior score is the sum of all responses; higher scores indicate more severe impairment. Functional assessments include the total functional capacity (TFC), the independence scale, and a checklist of common daily tasks; higher scores indicate better functioning.[[39]](#endnote-40)

The **Mini-Mental State Examination** (MMSE) is a brief standardized assessment of cognition in adults. It has many potential uses, which include screening for cognitive impairment, estimating the severity of cognitive impairment, and following the progression of cognitive changes over time.[[40]](#endnote-41)

**Family History Questionnaire** - Since its initial cogent description in 1872, it has been clear that HD has a strong hereditary contribution resulting in the generational transmission of the disease from parent to offspring, regardless of gender. Beginning in 1981 and through the collection of clinical and family history information and biological material (DNA) from HD families, largely in the Lake Maracaibo region of Venezuela, the gene mutation that causes HD was identified about a decade later (a novel gene containing a trinucleotide repeat that is expanded and unstable on Huntington's disease chromosomes).[[41]](#endnote-42)

There is a strong and consistent inverse relationship between the length of the CAG repeat (CAG_n_) mutation and the clinical onset of HD. However, CAG_n_ accounts for only about 60-70% of the variance in age at onset; other, as yet unidentified, extra-genetic and environmental factors influence age at onset and the cascade of pathogenic events resulting in the HD phenotype. Improved understanding of the pathogenesis of HD will require a systematic comparison of the relationship between the HD genetic mutation and its associated phenotype. To obtain valid correlations of genotype and phenotype, information should be collected in an accurate and standardized fashion beginning with documentation of disease transmission within the families of individuals participating in COHORT.

**Genotypic Evaluation** - An unstable, expanded CAG repeat within the coding region of the 4p16.3 *HD* geneError: Reference source not found explains many of the puzzling genetic features of the disorder, including the variable age at onset, the tendency for juvenile disease to be inherited from fathers, and the rare appearance of new mutations. DNA samples from COHORT subjects will permit genotyping of the CAG repeats in the mutant and normal HD alleles. While the number of CAG repeats can be measured with precision from DNA in blood samples, the emerging clinical precursors and their predictive value for the clinical onset of HD have not been prospectively measured or assessed. Thus, the unaccounted variance in determining when HD manifests may be appreciably larger or smaller depending on the standards, precision and specificity for assessing the clinical onset of HD.

Many studies have examined the relationship of CAG_n_ to age at onset for motor symptoms in HD[[42]](#endnote-43),[[43]](#endnote-44),[[44]](#endnote-45),[[45]](#endnote-46),[[46]](#endnote-47) and the correlation between repeat size and age at onset accounts for ~ 60-70% of the variation in age at onset. Recent studies suggest that the remaining variation in age at onset of HD is strongly heritable.[[47]](#endnote-48),[[48]](#endnote-49) These findings indicate that the onset of HD is substantially influenced by factors other than repeat size, and other modifier genes may determine the remaining variation in age at onset. Research on these other modifier genes continues to be conducted. For example, the UCHL1 gene, which encodes ubiquitin carboxyl-terminal hydrolase L1, was reported recently to influence age at onset of HD, with the S18Y polymorphisms accounting for 13% of the variance in age at onset in a case-and-control-study design.[[49]](#endnote-50) Several previous studies have reported that the glutamate receptor gene (GluR6; GRIK2) acts as an HD modifier.[[50]](#endnote-51),[[51]](#endnote-52) A particular TAA repeat allele in the 3’ UTR of GRIK2 was associated with younger age at onset of HD.[[52]](#endnote-53) When analyzed in conjunction with UCHL1, 7% of the variance in the age at onset of HD could be attributed to the GRIK2 genotype variation, 13% to UCHL1, and 16% to both polymorphismsError: Reference source not found). Chromosomal regions harboring additional modifier genes have been implicated by a recent genome linkage scan (the HD-MAPS study).Error: Reference source not found DNA samples from COHORT subjects will permit genotyping of polymorphisms in authenticated genes that have previously shown to modify the timing or presentation of disease features.

**Biological Specimen Repository** - Identification of additional genes that modify the pathogenic process in HD offers a direct route to validate targets for development of HD experimental therapeutics. To date, this has been carried out using age at onset of neurological (largely motor) signs as the variable phenotype, but it is clear that HD may display other phenotypic variability in disease expression, including sub-type characteristics of movement disorder (e.g., hyperkinetic, hypokinetic), psychiatric manifestations (e.g., depression, psychosis), cognitive impairment (e.g., impairment of executive function and/or immediate memory), rate of disease progression, degree of neuropathological involvement, pace and extent of neuroimaging abnormalities, etc. It is likely that additional phenotypic variations, characteristic of the pathogenic process, will be defined by more detailed analyses of gene expression, protein expression, metabolic measures, biomarkers searches, etc.

COHORT will provide a wide range of HD-associated phenotypes by which to identify modifier genes, and, by extension, environmental modifiers that may be similarly tested for an effect on HD phenotype. Initially, the phenotypes available will be derived from clinical assessments (UHDRS), but the collection of biological samples will permit the application of molecular phenotyping technologies at the levels of RNA, protein, metabolites and cultured cells. Collection of family history information and knowledge of familial relationships of COHORT will permit assessment of the variation of phenotypes within families and their degree of heritability. DNA samples from COHORT subjects will permit genotyping of the CAG repeats in the mutant and normal HD alleles and of polymorphisms elsewhere in the genome using standard approaches. The combination of phenotypic and genotypic information will permit analysis of relationships between individual polymorphisms and genes and the effect they have on modifying the nature, rate of progress and response to treatment of HD using standard genetic linkage and association strategies.

# DISCUSSION OF STUDY DESIGN

The COHORT study was designed to assemble a large descriptive research database for HD in order to study the natural history and progression of HD using systematic phenotypic description and correlations of genotype to phenotype. The consented provision of specified biological specimens will also provide qualified scientists with the material and correlative data to identify useful biomarkers for HD.

Wherein some longitudinal studies determine inclusion based upon geography or exposure, COHORT will determine inclusion based upon the inheritance of the HD gene. Theoretically, using the genome to determine the sample allows for the potential to include all HD families. Realistically, inclusion will be further defined by the geographic location of our HSG study sites. We have chosen to include all currently qualified and willing HSG sites in North America and Australia. Our HSG counterpart in Europe is currently developing a similar study.

Members of HD families, including spouses, where there is a confirmatory family history of HD will be asked to participate in COHORT. A confirmatory family history of HD is defined by the presence of either 1) a parent or sibling with clinically diagnosed features of HD or 2) a first-degree relative known to be a carrier of the HD gene through pre-symptomatic DNA testing. This will allow for four different groups of individuals to be enrolled: 1) those affected by HD, 2) those unaffected, but at 100% risk by nature of a positive gene test, 3) those unaffected, but at risk because of a parent, sibling, grandparent, or grandchild with HD, and 4) those unaffected, but at no risk because of a negative gene test or no family history (spouses).

The samples to be collected for the COHORT study will be used for a variety of different analyses. Cross-sectional analyses, designed to identify risk factors for particular disease symptoms, require modest sample sizes that can be estimated based on the size of the likely effect. Longitudinal analyses, designed to identify risk factors for disease progression, typically require larger sample sizes since the rate of follow-up can be quite variable and inconsistent between study subjects. The design of COHORT puts no limit on the sample size to be collected or a timeframe in which the study will be completed. It is intended that data and samples will be collected until an effective treatment for HD is established. The accrual of phenotypic, hereditary, genotypic, and extra-genic data will provide cumulative increases in statistical power to detect risk factors for relevant phenotypic characteristics or outcomes (e.g., progression of HD and ‘phenoconversion’ or clinical onset of disease) that can in turn improve the efficiency of therapeutic trials (e.g., to delay progression or phenoconversion). This is illustrated in the example and analysis in section VIII.

Genetic analyses designed to identify novel polymorphisms, which affect disease onset, clinical symptoms or disease progression, typically require even larger sample sizes, which vary with the design of the statistical analyses. For example, linkage analyses seek to identify chromosomal regions, which harbor genetic variants contributing to disease variability. Such studies typically employ data from hundreds of families. The exact number of families required for such analyses is affected by the magnitude of the genetic effect, the extent of genetic heterogeneity in the samples, and the density of the molecular markers being tested. To identify the specific genetic polymorphisms affecting disease risk or symptom variability it is essential to perform alternate analyses such as family based association analyses. This type of analysis can be relatively inefficient in its use of family resources, but is particularly resistant to possible sources of data bias such as sample stratification. Estimates of the required sample sizes must take into account the heterozygosity of the polymorphisms being tested, the genotypic effect (and/or relative risk conferred by the putative polymorphism), the genetic heterogeneity in the samples and the extent of linkage disequilibrium between the molecular markers being tested.

Inclusion of a biological specimen repository in COHORT evolved from discussions about the current unmet needs for HD research. Advances in understanding the pathogenesis of HD and the discovery of parallel biomarkers have largely been limited by the availability of suitably collected biological specimens and the availability of systematically collected and longitudinal data. The COHORT biological specimen repository will provide valuable research specimens for current and future scientific research aimed at developing useful biomarkers of HD. Utilizing the renewable resources found in the repository will further increase the availability of biological specimens for scientists who are prepared to carry out experiments utilizing the rich correlative data of phenotype, genotype and heredity that will be collected in COHORT.

Protecting confidentiality has been an overriding principle in the development and design of COHORT. The broad nature of the inclusion criteria creates a unique situation in which varying levels of concern regarding confidentiality will be apparent. COHORT was designed to provide a flexible situation in which subjects are given the ability to decide the procedures in which they will participate. The collection of clinical information and blood for CAG/genetic polymorphism analyses are the only required procedures in COHORT. Optional procedures include the collection of family history information and the storage of biological specimens in the repository.

While there is scientific merit for including both affected and unaffected individuals under the age of 18, only children with clinically diagnosed features of HD in the setting of a confirmatory family history or clinically diagnosed features of HD with a positive gene test will be eligible to participate in COHORT. Those children who are eligible to enroll will only participate in the phenotypic evaluation, which includes the neurological and physical examination, medical history, and the UHDRS 99. Children will only participate in the phenotypic evaluation until we are able to further evaluate the process by which these children will be able to ethically and safely participate in the other aspects of this study. Unaffected children will not be enrolled at this time because of concern over the potential psychological consequences of including this population in COHORT. In the future, this study may be amended to include these additional populations and procedures.

A system has been developed to ensure the protection of any identifiable data collected under COHORT. Although no subject names or personal identifying information will be used in the collection of clinical data or biological specimens, subject names and family member names will be collected in the family history component. This is crucial to research in genetic diseases because this information will be used to link families across participation in COHORT. Therefore, a system will be used to maintain rigid control over who has access to this information. Identifiable family history information will only be held at the site and at Indiana University, where the pedigrees will be created. Plots of the family history information will be generated without any identifying family information (such as names) and this database will not be connected to the other databases used in this study.

A stringent arrangement has been created that protects confidentiality by maintaining different databases to hold the clinical data, family history data, and the biological specimens. Each database will be held in a different geographic location without any direct electronic connections between the databases. A common subject identification number will be maintained in each database to allow for deidentified data to be combined across databases for analysis.

As part of our efforts to ensure protection of genetic, family history, and clinical information, a Certificate of Confidentiality has been obtained from the Department of Health and Human Services. This certificate will be used to enhance the confidentiality for subjects in COHORT. The extra layer of protection is a necessity considering the potential for inadvertent or unexpected disclosure of gene or clinical status, which could cause negative employment consequences, difficulty obtaining insurance, and social or familial consequences.

# CHARACTERISTICS OF THE RESEARCH POPULATION

- 1. **Number of Subjects**

This study will include as many subjects who are eligible and willing to participate. We expect that thousands of individuals may participate.

- 1. **Gender of Subjects**

Both males and females will be included in this study.

- 1. **Age of Subjects**

Individuals 18 years of age and older will be asked to participate in all aspects of the study. For individuals under the age of 18, only those with clinically diagnosed features of HD in the setting of a confirmatory family history or clinically diagnosed features of HD with a positive gene test will be eligible to participate. Subjects under the age of 18 will only participate in the phenotypic evaluation, which includes medical history, neurological and physical examination, and the UHDRS 99. It will be left up to the discretion of the site investigator to determine if a subject under the age of 18 is able to complete the cognitive assessment, functional assessment, independence scale, and functional capacity in the UHDRS 99.

If a child turns 18 while participating in COHORT, he or she will be required to provide written consent to continue participation in the study.

- 1. **Racial and Ethnic Origin**

There are no restrictions on ethnicity or race. Past experience in HD research has shown an underlying lower prevalence of HD in non-Caucasian populations, although there are no definite racial predispositions and the disease is found throughout the world.

- 1. **Inclusion Criteria**

For those 18 years of age and older, there are four different types of individuals who may be eligible to participate in COHORT:

1. An individual with clinically diagnosed features of HD in the setting of a confirmatory family history or an individual known to carry the HD gene as confirmed by DNA testing (individual is defined as the ‘proband’).
2. A first-degree relative (parents, siblings, or children) of an individual with clinically diagnosed features of HD in the setting of a confirmatory family history or of an individual known to carry the HD gene as confirmed by DNA testing.
3. A grandparent or grandchild of an individual participating in COHORT who meets criterion 1.
4. A HD family member who has no risk for HD due to no family history (spouses of proband only) or negative gene testing. This individual must have an affected or gene positive spouse or family member participating in COHORT to be included.

For individuals under the age of 18, only those with clinically diagnosed features of HD in the setting of a confirmatory family history or clinically diagnosed features of HD with a positive gene test will be eligible to participate in COHORT (individual may be defined as the ‘proband’).

A confirmatory family history of HD is defined by the presence of either: 1) a parent or sibling with clinically diagnosed features of HD or 2) a first-degree relative (parent, sibling, child) known to be a carrier of the HD gene through pre-symptomatic DNA testing.

Subjects may be male or female. All subjects must be able to provide consent for themselves, have a parent/guardian who can provide parental permission, or have an authorized representative who can provide consent.

- 1. **Exclusion Criteria**

Anyone who does not fit the criteria stated above.

- 1. **Vulnerable Subjects/Subject Capacity**

Children and mentally compromised individuals will be included in this research study.

Only children with clinically diagnosed features of HD in the setting of a confirmatory family history of HD or clinically diagnosed features of HD with a positive gene test (performed as part of clinical care) will be eligible to participate. Children will be included in this study because we would like to have a database that reflects the continuum of illness and all populations affected by it including juvenile HD. They present with a spectrum of symptoms unique to their age of onset. Their incidence is approximately 10% of all affected by HD. Opportunities to better understand this group and treat it are thus important. Parental permission will be obtained from one parent/guardian for each subject under the age of 18 who participates in this study. In addition to this verbal or written assent will be obtained when appropriate. Guidelines for obtaining assent include:

- Children under the age of 7, only parental permission will be obtained.
- Children 7 to 12 years of age, parental permission with verbal assent will be obtained.
- Children 13 to 17 years of age, parental permission with written assent will be obtained.

Any subject who begins participation in this study as a minor (under the age of 18) will be required to provide written consent to participate in this study after turning 18 years of age.

Individuals with HD whose disease has progressed to the point of mental incapacity may be enrolled in this study. The site investigator will determine mental incapacity at the baseline visit. Mentally compromised individuals will be asked to participate with the consent of an authorized representative (see below for additional information).

# SUBJECT IDENTIFICATION, RECRUITMENT AND CONSENT/ASSENT

- 1. **Method of Subject Identification and Recruitment**

The research staff at the site will recruit potentially eligible subjects and inquire as to their willingness to participate in this study. These individuals may be recruited through clinical practice, support groups, advocacy newsletters, etc. All methods of recruitment will be reviewed and approved by the Institutional Review Board before use. A subject newsletter will be provided at the initial visit and at each subsequent yearly follow up visit to subjects who are participating in COHORT. This newsletter will be provided for informational purposes, but may also be used for recruitment purposes. The site coordinator may provide it to individuals who are considering participation in the study or for subject’s to distribute to family members who may be interested in participation.

- 1. **Process of Consent**

The research staff at the site will seek consent from any eligible subject willing to participate. At the time of the subject’s visit an explanation of the study and a copy of the consent will be provided. The opportunity to read the consent will be given and questions answered by the research staff. The subject will be given the option to take the consent form home to discuss with family members, and consent will be obtained at a future visit. Participation in the study will not be pursued if the individual declines interest, unless the individual agrees to future discussion.

If a subject with mental incapacity is approached for enrollment, the research staff will seek consent from that subject’s authorized representative. An authorized representative would be determined consistent with state law, and may be defined as an individual with guardianship, the subject’s next-of-kin, a relative, or a long-term caregiver/significant other. This person must be mentally cognizant and be able to understand the procedures, risks, and benefits involved with the study. If a health care proxy has been determined and consenting for research studies is within the scope of the proxy’s delegated responsibilities, the health care proxy may provide consent for this study. At the time of the subject’s visit, an explanation of the study and a copy of the consent will be provided to both the subject and the authorized representative. Opportunity to read the consent will be given and questions answered by the research staff. The subject/authorized representative will be given the option to take the consent form home with them to discuss with family members and consent obtained at a future visit. Although the authorized representative will be officially providing consent for the subject to participate, the subject must also agree to participate.

Subjects with HD may lose mental capacity to provide continued consent during the course of this study because of the long-term nature of the study. Therefore, it will be recommended to subjects who have been diagnosed with HD to appoint a research proxy to aid in the decision making process for continued participation. Subjects will be encouraged to discuss their future study participation wishes with the research proxy. Those individuals who have already been diagnosed with HD at enrollment may appoint a research proxy at the time of enrollment. Those individuals who are diagnosed with HD while participating in this study will appoint a research proxy at the time of diagnosis or at the next study visit. During the study, the site investigator will determine whether or not the subject has diminished mental capacity and will determine when the research proxy needs to be contacted to act on behalf of the subject. The site investigator will contact the research proxy and request that he/she attend the next scheduled study visit.

- 1. **Documentation of Consent/Assent**

Signed consent/assent/parental permission forms will be stored in a designated location at the site. A signed copy of the consent/assent/parental permission will be provided to the subject/parent/guardian and, if applicable, their authorized representative.

- 1. **Consent/Assent forms**

The following forms will be used during this study

- - Consent form (adult participation) – obtained prior to subject enrollment in the study
  - Consent form Addendum (adult participation) – used throughout the study if a subject changes his/her mind about participation in an optional aspect of the study
  - Parental Permission form – obtained prior to a child’s enrollment in the study.
  - Parental Permission Addendum – used throughout the study if a parent/guardian changes his/her mind about participation in contact between visits or future contact.
  - Assent form - obtained prior to the enrollment of a child (always obtained in conjunction with parental permission)
  - Identification of Research Proxy
  1. **Waiver of Consent/Waiver of Authorization**

If a subject chooses to participate in the family history portion of COHORT, the subject will provide his or her full name and the names, basic demographic information, and HD status of their family members. Consent and authorization will be obtained by the subject to provide his or her name and information, but a waiver of consent and a wavier of HIPAA Authorization are needed to collect information about the family members. As per 45CFR46.116(d) 1-4, an IRB may waive the requirements to obtain informed consent if the following four elements are properly satisfied.

**Waiver of Consent**

1. The research involves no more than minimal risk to the subjects.

Names will only be used within the database at Indiana University to link families across participation in the study and to create pedigrees. The pedigrees that are created from this database will contain no identifying information and no names will be used as a method of recruitment.

1. The waiver or alteration will not adversely affect the rights and welfare of the subjects.

A system has been developed to maintain rigid control over access to identifiable data and a Certificate of Confidentiality will be obtained to provide additional protection to ensure the rights of the welfare of the subjects. Identifying information will only be stored at the site and at Indiana University. Sites will be required to store hard copies of the questionnaires in areas that are locked 24 hours per day. Indiana has extensive experience in conducting genetic and pedigree research studies where family history is collected. Indiana’s computer system and the protections in place to protect confidentiality are more thoroughly described in section VII (b).

1. The research could not practicably be carried out without the waiver or alteration.

It is crucial for conducting genetic research to allow families to be connected across participation in COHORT. The ability to conduct this study in different geographic locations allows for family members to participate all over the country and all over the world, without knowledge of other family member’s participation. Collecting family member names will allow for connections to be made across families and across sites, while protecting the confidentiality of the individuals participating in the COHORT. The genetic variation that causes Huntington’s disease increases the necessity for genetic research including pedigrees to be conducted. This allows for phenotypic and genotypic comparisons to be made across families to learn more about Huntington’s disease and to have the ability to accurately document disease transmission within families.

There is no limit on the number of individuals who can enroll in this study. Therefore, it is possible that thousands of individuals will participate in the family history aspect of the study. It would be impossible to obtain consent from each family member of the subject to be included in the family history database. If the pedigrees are only half complete, the scientific validity and power with which we will be able to use these pedigrees to determine the potential causes for genetic variation within families will be severely undermined.

1. Whenever appropriate, the subjects will be provided with additional pertinent information after participation.

The family members will never be contacted by any of the research staff based on the information collected on the family history questionnaire. Therefore the family members will not be provided with any information.

As per 45CFR164.512(i)2(ii), the IRB or privacy board can determine that authorization may be waived if the following elements are properly satisfied.

**Waiver of Authorization**

1. The use or disclosure of protected health information (PHI) involves no more than minimal risk to the individuals.

The use of this information at the site and the disclosure of this information to Indiana University is no more than minimal risk to the family members, as the information will be held in the strictest of confidence at both sites. The names will only be used within the scope of the database to connect participation across families and any pedigrees created will not contain identifying information. Names will not be used as a method of recruitment

1. The alteration or waiver will not adversely affect the privacy rights and the welfare of the individuals.

A system has been developed to maintain rigid control over access to identifiable data and a Certificate of Confidentiality will be obtained to provide additional protection to ensure the rights of the welfare of the subjects. This information will only be stored at the site and at Indiana University. Sites will be required to store hard copies of the questionnaires in areas that are locked 24 hours per day. Indiana has extensive experience in conducting genetic and pedigree research studies where family history is collected. Indiana’s computer system and the protections in place to protect confidentiality are more thoroughly described in section VII (b).

1. The research could not practicably be conducted without the alteration or waiver.

There is no limit on the number of individuals who can enroll in this study. Therefore, it is possible that thousands of individuals will participate in the family history aspect of the study. It would be impossible to obtain authorization from each family member of the subject to be included in the family history database. If the pedigrees are only half complete, the scientific validity and power with which we will be able to use these pedigrees to determine the potential causes for genetic variation within families will be severely undermined.

1. The research could not practicably be conducted without access to and use of the protected health information.

It is crucial for conducting the genetic research planned in COHORT to connect families across participation in the study. Collecting PHI will identify those with HD and those without HD, which will be essential in relating phenotypes and genotypes across families. The genetic variation that causes Huntington’s disease increases the necessity for genetic research including pedigrees to be conducted in order to learn more about Huntington’s disease and to have the ability to accurately document disease transmission within families.

1. The privacy risks to individuals whose protected health information is to be used or disclosed are reasonable in relation to the anticipated benefits if any to the individuals, and the importance of the knowledge that may reasonably be expected to result from the research.

The use of PHI in this study will be invaluable to the planned genetic research. It is the hope that this genetic research will identify genes that may modify the pathogenic process of HD and may offer potential targets for the development of HD experimental therapeutics.

1. There is an adequate plan to protect the identifiers from improper use and disclosure.

The identifiers will only be stored at the site and at Indiana University. Sites will be required to store hard copies of the questionnaires in areas that are locked 24 hours per day. Indiana’s computer system and the protections in place to protect confidentiality are more thoroughly described in section VII (b). Indiana has extensive experience in conducting genetic and pedigree research studies where family history is collected. Names will only be used within the database to link families across participation in the study and any pedigrees that are created from the database will not contain identifying information. Names will not be used as a method of recruitment.

1. There is an adequate plan to destroy the identifiers at the earliest opportunity consistent with conduct of the research, unless there is a health or research justification for retaining the identifiers, or such retention is otherwise required by law.

When this study is concluded, defined by the final subject visit and completion of analysis, the names of all subjects will be removed from the computer database at Indiana and replaced with a unique identifier that is not associated with the name of the individual.

1. There are adequate written assurances that the protected health information will not be reused or disclosed to any other person or entity, except as required by law, for authorized oversight of the research project, or for other research for which the use or disclosure of protected health information would be permitted by this subpart.

The PHI collected under this study will only be used under the context of the COHORT study or the COHORT repository. All researchers who request samples from the repository will be required to sign an assurance where the researcher agrees to never attempt to identify or contact the subject.

# VI. STUDY PROCEDURES

- - - - 1. **Methods and Procedures**

Study visits will take place yearly and may occur at the time of the subject’s standard of care visit. At the **baseline visit** the following procedures will take place:

- - Obtain written informed consent/parental permission/assent
  - Review of inclusion/exclusion criteria
  - Collect demographic information.
  - Research staff will ask the subject questions and record information regarding medical history and concomitant medications.
  - Complete physical examination that includes the measurement of height, weight, and vital signs.
  - Complete neurological examination.
  - Unified Huntington’s Disease Rating Scale 99 (UHDRS 99) For subjects under the age of 18, it will be left up to the discretion of the site investigator to determine if the child is able to complete the cognitive assessment, functional assessment, independence scale, and functional capacity of the UHDRS 99.
  - Mini-Mental State Examination (MMSE) will be performed on subjects who are 18 years of age and older.
- Blood sample (10 ml) colleted for CAG genotyping and genotyping for other genetic polymorphisms (only offered to subjects who are 18 years of age and older).

The following **optional** procedures will be offered to the subject at the **baseline visit**:

##### Contact between study visits

- Family History Questionnaire (FHQ) (only offered to subjects who are 18 years of age and older)
- Blood (20 ml) collected for storage in a Specimen Repository for future HD research (only offered to subjects who are 18 years of age and older). Although consent for urine collection maybe obtained at the baseline visit, urine will be collected at a subsequent yearly visit.
- Future contact regarding research studies

The following will take place at the subsequent **yearly** visits:

- - Unified Huntington’s Disease Rating Scale 99 (UHDRS 99) - For subjects under the age of 18, it will be left up to the discretion of the site investigator to determine if the child is able to complete the cognitive assessment, functional assessment, independence scale, and functional capacity of the UHDRS 99.
  - Mini-Mental State Examination (MMSE) will be performed on subjects who are 18 years of age and older.
  - Brief medical evaluation including height, weight and vital signs.
  - Information collected about medical and mental health events, which have occurred since the last visit and changes in concomitant medications.
  - Subjects who are 18 years of age and older will be given the option to re-evaluate participation in any of the optional procedures. Throughout the study, if a subject changes his or her mind about participation in an optional aspect of the study, this decision will be documented using the consent addendum.
  - If the subject agreed to participate in the FHQ they will be asked to provide information about new deaths and new HD onsets within the family affected by HD.
- If the subject agreed to have blood stored in the specimen repository, blood (20 ml) will be collected.
- If the subject agreed to have urine stored in the specimen repository, urine (50 ml) will be collected at the 2^nd^, 3^rd^, or 4^th^ yearly visit. The sites and the subjects will be notified at which visit this will be collected.

If a subject dies while participating in COHORT the circumstances of a subject’s death will be assessed and recorded. If an autopsy is performed as part of clinical care, the site may try to obtain information from the autopsy for the COHORT study. If this takes place, the subject’s next of kin will be contacted to provide authorization. The authorization form will be reviewed and approved by the site’s IRB or Privacy Board before authorization is obtained.

**COHORT Schedule of Activities**

| **Study Procedure** | **Baseline**  **Visit**  **(Visit BL)** | **Subsequent Yearly Visits**  **(1^st^ annual visit**  **will be Visit 1)** |
| --- | --- | --- |
| Informed consent | X |  |
| Review Inclusion/Exclusion | X |  |
| Medical history | X |  |
| Complete physical and neurological examination | X |  |
| Brief Medical Evaluation^1^ |  | X |
| Medical and Mental Health Event Review |  | X |
| Concomitant Medication Review | X | X |
| UHDRS 99^2^ | X | X |
| MMSE^3^ | X | X |
| Blood draw for CAG genotyping/genotyping for other genetic polymorphisms^4^ | X |  |
| Family History Questionnaire^5^ | X | X^6^ |
| Blood draw for Repository^5^ | X | X |
| Urine for Repository^5^ |  | X^7^ |

^1^ Brief Medical Evaluation includes measurement of height, weight and vital signs.

^2^ For subjects under the age of 18, it will be left up to the discretion of the site investigator to determine if the child is able to complete the cognitive assessment, functional assessment, independence scale, and functional capacity.

^3^ MMSE will be only performed on subjects who are 18 years of age and older.

^4^ Blood for CAG genotyping/genotyping for other genetic polymorphisms will be only performed on subjects who are 18 years of age and older.

^5^ These are optional procedures for subjects who are 18 years of age and older.

^6^ Subjects will only be asked about new deaths and HD onsets.

^7^ Urine Samples will be collected at either the 2^nd^, 3^rd^, or 4^th^ yearly visit. The sites and the subjects will be notified at which visit this will be collected.

**b) Procedure Descriptions**

**Unified Huntington’s Disease Rating Scale 99 (UHDRS 99)**

The Unified Huntington’s Disease Rating Scale 99 (UHDRS 99) will be used to examine the clinical aspects of HD. The UHDRS 99 is a comprehensive evaluation that consists of four parts: motor function, cognition, behavior, and functional capacity. The total UHDRS 99 takes approximately 1 hour to complete.

The UHDRS 99 will assess a variety of physical and cognitive functions. This includes evaluations of movement (eye, finger, and hand movements and walking), behavior, and functional capacity (questions about mood, behavior, and ability to perform day-to-day activities). The UHDRS 99 also includes tests of memory and attention. The individual who comes to the research visit with the subject may be asked to corroborate or supplement the subject’s responses during the evaluation. This individual may be able to provide information that may not occur to the person with HD because of impaired recall caused by HD. This individual may be the identified research proxy, but it is not required. For subjects under the age of 18, it will be left up to the discretion of the site investigator to determine if the child is able to complete the cognitive assessment, functional assessment, independence scale, and functional capacity.

To maintain consistency of the data collected for COHORT, the site must have the same individual(s) perform the UHDRS 99 for a particular subject from year to year.

- - - 1. **Mini-Mental State Examination**

The Mini-Mental State Examination (MMSE) will be used as a cognitive assessment throughout the study. This examination takes between 5 and 15 minutes to complete and will be conducted at every visit on those subject who are 18 years of age and older.

The exam asks basic questions about cognitive function including registration, attention and calculation, recall, and language.

- - - 1. **CAG Genotyping/Genotyping for other Genetic Polymorphisms**

All subjects 18 years of age and older will have a blood specimen (10ml) collected at the baseline visit. DNA will be extracted from this blood specimen to measure the length of the CAG polymorphism (genotype the HD CAGn) and to genotype other authenticated genetic polymorphisms previously shown to modify timing or expression of disease features. The DNA genotyping for the HD CAGn and genotyping for authenticated polymorphisms in other genes previously shown to modify timing or expression of disease features will be performed in a research lab and therefore the results are experimental data. Under no circumstances will the results be reported to the sites or to the subjects. If a subject decides to seek DNA testing for the HD CAGn while participating in this study, he/she may do so through the available HD predictive testing centers.

Coriell Institute for Medical Research will be the company processing and storing blood and urine specimens for the COHORT study. The following process will be utilized for the collection and processing for CAG genotyping and genotyping for other authenticated genetic polymorphisms:

- One tube of blood (10 ml) will be collected and shipped by overnight courier with the site number and subject number for identification purposes.
- Coriell will assign a unique identifier to the sample.
- DNA will be obtained from the blood using a salting out procedure.
- Routine quality control studies will be conducted to estimate the quality and integrity of the DNA
- All activities and testing will be documented by Coriell.

The DNA will then be sent to a laboratory at Massachusetts General Hospital where the CAG genotyping and genotyping for authenticated modifier genes previously shown to modify timing or expression of disease features will be conducted. CAG and polymorphism genotyping will be performed at the DNA Laboratory (Molecular Neurogenetics Unit, Massachusetts General Hospital) under the supervision of Marcy MacDonald, PhD who, with her collaborators identified the CAG expansion of the mutant HD gene and who has considerable experience with the analytic technique. DNA genotyping and genotyping for authenticated modifier genes will be performed on coded DNA samples. For the purposes of genotyping, MGH DNA laboratory will receive DNA samples identified only by the Coriell identification number assigned by Coriell Institute for Medical Research.

- - - 1. **Contact Between Study Visits**

###### Confidentiality is a major concern for most individuals who have HD or are at risk of having HD. Therefore, subjects will be given the option of allowing the study staff to contact them between study visits to collect additional information or to provide updates regarding the study. If a subject agrees, he/she will receive information about the study as it progresses and may be contacted between visits for additional clarifications. Confidentiality is of utmost importance, but to maintain the validity of the study, retention of subjects longitudinally is crucial. Although subjects will be given the option of receiving information as stated above, they will not be given the option for contact regarding study appointments. The study staff will work with each subject to set up the best method for contact between study visits for appointment confirmation and to reschedule any missed appointments.

- - - 1. **Family History Questionnaire (FHQ)**

Any subject 18 years of age and older with a family history of Huntington’s disease will be asked to complete the FHQ. This portion of the study is completely optional. The primary questionnaire will be completed only once during the study. The subject may complete the FHQ at the first visit or they may choose to complete the questionnaire at a subsequent yearly visit.

The subject will be given the option of completing the questionnaire during the study visit or of taking the questionnaire home and mailing it back to the site with a stamped, addressed envelope.. If the completed questionnaire is not received within one month of the study visit, the site will follow-up with the subject.

The FHQ will only collect information about the side of the family that is affected by Huntington’s disease. The FHQ will collect information about the spouse, parents, grandparents, children, siblings, aunts, uncles, nieces, and nephews. The questionnaire will collect name, sex, birth date, and date of death, if applicable. For those family members affected with HD, it will also collect information regarding age of HD diagnosis and if a physician diagnosed HD. This questionnaire may take as long as 90 minutes to complete. The extended family members identified on the questionnaire would never be contacted by anyone working on the study. The information collected will only be used to identify and link families in which multiple members are participating in COHORT. This linkage across families will allow for data to be used in analyses designed to identify genetic polymorphisms affecting variability in disease onset, clinical symptoms, and disease progression.

If the subject agrees to participate in the FHQ they will be asked at each subsequent visit to complete an update to the questionnaire providing information about new deaths and new HD onsets that have occurred on the side of the family affected by HD.

**Removal of Family History Data**

Subjects can request to have information they provided be removed from the COHORT family history database at Indiana University School of Medicine. In order to request the removal of family history data, the subject must complete the “Removal of Information from the COHORT Family History Database” form to indicate what they would like removed:

- 1. Remove all information the subject provided (personal information and information about the subject’s family) – Any information the subject provided will be removed from the database and if any family member provides information about the subject in the future this will also be removed.
  2. Remove any personal information about the subject – The subject did not agree to participate in the family history database and wants to ensure that their data will not be included. If a family member provides information about the subject in the future this will be removed.
  3. Remove specific information the subject provided – The subject wants to specify the information that will be removed from the family history database. The subject will highlight the specific information to be removed on the FHQ or Annual Update and attach this to the ““Removal of Information from the COHORT Family History Database.”

The ““Removal of Information from the COHORT Family History Database” will be submitted to the Department of Medical and Molecular Genetics at Indiana University School of Medicine where the staff will facilitate the removal of the information from the COHORT family history database. In some cases the gender of the subject must remain in the database, particularly, if descendents of the subject wish to continue their participation in the family history database. Indiana will keep a list of these names and will periodically check the database to ensure that they are consistently removed from the database.

- - - 1. **Specimen Repository**

***Blood*** – Only subjects 18 years of age and older will be asked to participate. This portion of the study is completely optional. If the subject agrees to have blood samples stored in the specimen repository, 2 tubes of blood (total volume - 20 ml) will be collected at each visit. The subject may choose to participate at the baseline visit or at any of the subsequent yearly visits.

The blood will be sent to Coriell Institute for Medical Research and processed for storage in the COHORT specimen repository. This blood will used to store plasma, lymphocytes, and lymphoblastoid cell lines for future HD research. Cell lines will only be created one time. If for any reason the cell line fails, frozen lymphocytes can be used to make another cell line. When the cell line is created, DNA will be extracted from the cell line and it will be sent to MGH to be used in the genotyping for the HD CAGn and genotyping for authenticated polymorphisms in other genes previously shown to modify timing or expression of disease features. For the purposes of genotyping, MGH will receive DNA from the cell line that is identified only by the Coriell identification number assigned by Coriell Institute for Medical Research. The other portion of the lymphoblastoid cell line will be stored for future research in Huntington’s disease. The following process will be performed for the creation of samples for the repository:

- Blood will be collected and shipped by overnight courier with the site number and subject number for identification purposes.
- Coriell will assign a unique identifier to the sample.
- Samples will be processed including appropriate testing for viability and contamination.
- All activities and testing will be documented by Coriell.

***Urine*** – Only subjects 18 years of age and older will be asked to participate. This portion of the study is completely optional. If the subject agrees to have urine samples stored in the specimen repository, urine (50 ml) will be collected at either the 2^nd^, 3^rd^, or 4^th^ yearly visit. The sites and the subjects will be notified at which visit this will be collected.

Coriell will oversee the repository based on strict guidelines set forth by the COHORT Steering Committee. For additional information, the COHORT Data & Specimen Repository Access Policy and Procedures is located in the Appendix.

- - - 1. **Future Contact Regarding Research Studies**

###### Subjects will be given the option of allowing the site research staff to maintain their name and contact information so that the site research staff may contact them regarding future participation in HD studies. This information will be kept confidential and will be stored at the site.

1. **Costs to the Subject**

Subjects will incur no cost for participation in this study.

## Payment

Subjects will receive no payment for participation in this study.

## Data Management, Data Storage and Confidentiality

***Data Management***

**Clinical Trials Coordination Center** - The Clinical Trials Coordination Center (CTCC) and the University of Rochester Biostatistics Center will work together to serve three main functions: 1) Project Coordination, 2) Data Management, and 3) Data Analysis and Interpretation. The CTCC is principally responsible for the first two activities and the Biostatistics Center for the latter.

The CTCC will work in collaboration with the PI and Co-PIs to accomplish all the tasks necessary for preparation and implementation of the project, including the drafting and refinement of the study protocol, development of the model consents/assents, assisting in communications with the Steering Committee, developing project timelines, compiling required documents (IRB approvals of protocol and consent), overseeing development/design of CRFs, preparing the detailed operations manual which outlines all the tasks required for study implementation at the sites, overseeing the printing and distribution of documents, updating the protocol and operations manual as needed, updating regulatory files for the study overall, as well as supervising the timely renewals of IRB approvals at the sites. The CTCC will assist the PI in overseeing the site activities, working with the PI to finalize budgets for the central coordination and site activities, and in establishing subcontracts with all sites and other research units.

Throughout the study, the CTCC will be responsible for overseeing enrollments including real-time monitoring of enrollments, conveying information to the PI, Steering Committee and sites; fielding and replying to site protocol inquiries; and consulting as necessary with the PI. The CTCC will work in concert with Coriell Institute for Medical Research, Massachusetts General Hospital, and Indiana University Medical Center to set up the required processes to properly coordinate the activities of the study. This includes overseeing the appropriate transfer of data between these organizations.

The Data Management staff at the CTCC will be responsible for all aspects of data procedures, from the design of instruments used to collect data through the delivery of an accurate and timely database to the HSG Biostatistics Center. In conjunction with the PI and Project Coordinator, the data management staff at the CTCC will design, print, and distribute the CRFs that will capture all the data collected as part of the protocol.

Designated site personnel will enter the enrolled subject’s data on multiple-part no-carbon-required (NCR) CRFs during each study visit. The site coordinator will send a package of completed CRFs to the CTCC. A Data Control Clerk will review the forms and accompanying inventory sheet when the forms are received. CRFs are packaged for transmission to an off site vendor, DATROSE Inc., for data entry by a double keying technique. CRFs will then be returned from data entry with an electronic data set, which are checked to ensure consistency between the CRFs and the electronic records. These electronic records will then be loaded into a data table via SAS on the data server (Unix). A database will be created at the CTCC to store the data collected from the CRFs. The data will be reviewed and customized error-checking programs will be used to check the data. Standard Operating Procedures (SOPs) are in place for all data management activities at the CTCC**.**

# New CTCC Data Management Initiatives - While the paper-based data management process has been successfully utilized in all previous CTCC studies, the CTCC is currently implementing an advanced set of computer systems that will provide for the efficient capture, processing, analysis, and sharing of clinical data. The core of the new capabilities is an integrated Clinical Data Management System (CDMS) providing efficient data capture and processing from both paper and paperless sources (electronic data capture - EDC). This system is expected to be operational in March 2005 and will be utilized for the COHORT trial.

Study sites for COHORT will initially and predominantly utilize paper CRFs. As the study progresses and new sites are added, sites with the capability may be transitioned to the use of electronic CRFs (eCRFs) for entry of data into the CDMS. These eCRFs will collect data identical to the paper CRFs, and the eCRFs along with all electronic edit checks will be fully validated prior to the use of EDC at any site.

The new clinical data management system has the technical features that meet guidelines set by the US Federal Regulations, 21 CFR Part 11 - Electronic Records for remote data entry using electronic case report forms (eCRFs). Authorized study personnel will each be granted access to the electronic data capture tool via provision of a unique User ID/Password combination that will limit access to enter and view data specifically for subjects enrolled at their site. Sites utilizing eCRFs will initially enter all data into the subject’s medical chart and/or onto source documentation forms (SDFs) prior to entering data into the eCRFs via computer stations connected remotely to a central server through an Internet browser. The data entered from the eCRFs will be securely transmitted (using Secure Socket Layer 128 bit encryption algorithm) upon entry via the Internet connection to a central database stored on a secure server located at the Clinical Trials Coordination Center at the University of Rochester (Rochester, NY). Remote data entry will allow for near real-time data management, allowing for more efficient data collection and management. To minimize database errors, data will be checked and validated, upon entry by site personnel with the appropriate access rights while logging of all database activities (data entered / modified by whom and when) is maintained. Additionally, queries can be generated and resolved immediately through controlled range of responses, automated edit checks and an online query management facility.

For sites utilizing EDC, paper-based study forms will also be available to serve as back-up documents in the event of EDC failure. Source documentation forms (SDFs) per study visit will be provided in binders for each subject. Sites will also be responsible for maintaining other medical records obtained by site investigator and/or coordinator as primary source of data. Upon completion of a subject’s visit or the study, sites can opt to print the completed eCRFs depicting the data that was entered if so desired. At the conclusion of the study, a CD containing a PDF (portable document format) file depicting completed eCRFs for each site’s will be provided for record keeping.

**Indiana University** - The Department of Medical and Molecular Genetics at Indiana University School of Medicine will be responsible for the creation and data management of the Family History Questionnaire, including data entry into a secure computer system. Indiana will assign their own identification number for each subject that will be held only in the Indiana database. Indiana will also assign families a family identification number, which will link family members in their database. The family history data, obtained from the FHQ, are entered and stored in a multi-user relational database, Microsoft Access. The data from this database can be used to create all family structure files needed for genetic analyses. Key fields are automatically indexed and coded to enable fast and efficient searches through very large databases. Validation and integrity checks are performed interactively to minimize data entry and reduce inconsistencies. Data are easily exported to other software packages for statistical and genetic analysis, presentation or correspondence.

Pedigree plots of the family structure are performed using the Medical Genetics Data and Acquisition and Transfer System (MEGADATS) program, which was specifically designed, at Indiana University, to display and print complex pedigrees. Information entered from the FHQ is used to produce a pedigree plot and corresponding pedigree list. These data, when combined with clinical information, yield an extremely useful visual presentation of the variability within families.

Regular data transfers will take place between the CTCC and Indiana to confirm that Indiana has received all expected FHQs. A CRF submitted by the site will indicate if a subject has consented to the FHQ, when they consented and when the document was sent to Indiana. This information will be crosschecked with the FHQs received by Indiana to determine if Indiana is missing any FHQs. Indiana will then contact the site to resolve any issues regarding missing questionnaires.

All queries for the Family History Questionnaire will be generated and resolved by Indiana University through the study site. Discrepancy reports are routinely generated which identify inconsistent data such as children born prior to their parents, parents of the same gender, etc. The discrepancy reports will be sent to each site on a regular basis. The site will then contact the subject between visits (if the subjects has consented to this) to clarify all apparent errors. If the subject has not consented to be contacted between visits, the site will have to wait to resolve the query at the next visit. The site will then send back to Indiana University a log clarifying how each discrepancy is to be resolved. Reminders will be sent to sites on a regular basis to remind them of outstanding discrepancies.

Massachusetts General Hospital DNA Genotyping Laboratory- The process for CAG genotyping and genotyping for authenticated polymorphisms in other genes previously shown to modify timing or expression of disease features will proceed in the following sequence. MGH will receive the DNA samples from Coriell with only the Coriell identification number. If the subject consented to storing samples in the repository, DNA from the cell line created from the blood sample will be sent to MGH and utilized for genotyping. These cell lines will be coded and identified only by the Coriell identification number assigned by Coriell Institute for Medical Research. The samples will be reviewed, scanned and logged into an Excel database unique for the COHORT study. The database used for COHORT is on an 'isolated' PC computer that has double password protection, and which is not networked. Back-ups are kept in Dr. MacDonald's office, which is locked at all times. The results are read into the Excel database and checked for accuracy by Dr. MacDonald. The results are stored in a locked room and except for the Coriell identification code are not labeled in any other way. The results from each subject will be sent to the HSG Biostatistics Center where the results will be added to the clinical data to create a single dataset for analysis.

**Coriell Institute for Medical Research** - The COHORT Repository will be held at Coriell Institute for Medical Research, which will be responsible for receiving and cataloging all biological specimens. The Coriell database will store the subject identification number, the Coriell identification number, age at time of collection, gender, whether or not phenotypic and family history data is available for a particular sample, and the category the subject met for inclusion into the study. Researchers will have the ability to request any phenotypic information that has been collected in the study. . If a request is made for phenotypic information with specimens in the repository, the subject identification number will be used to link samples, phenotypic and family history information together. The correlated information will be compiled and de-identified, then will be sent to the requesting researcher. Very specific requests or requests that require very individualized data, such as sibling pairs or family relations, will be handled with Indiana and the CTCC on an individual basis.

Regular data transfers will take place between the CTCC and Coriell to confirm that Coriell has received all expected samples. A CRF submitted by the site will indicate if a subject has consented to the specimen repository and when they consented. This information will be crosschecked with specimens that Coriell has to determine if any specimens are missing.

***Data Storage and Confidentiality***

The CTCC will initially provide each site with a block of 100 subject identification numbers. The site will sequentially assign these numbers as subjects are prescreened for participation in the study. The CTCC will not have any access to subject names. At the conclusion of the baseline visit, the site will log on to the new clinical data management system using a unique User ID/Password combination that will limit access to enter and view data specifically for subjects at their site. While in this system the individual will use the electronic data capture tool to indicate that the subject has enrolled into the study. All source documents and case report forms will be labeled with the subject identification number. The subject will also be identified with a CTCC Unique Identification number. The CTCC Unique ID system was designed and implemented in response to the desired ability to track individual subjects across multiple studies without storing any personally identifiable information.

The system uses an algorithm, which takes as input nine data elements (last name, first name, gender, day of birth, month of birth, year of birth, city of birth, country of birth, and mother’s maiden name) and produces as output a "fingerprint" of the input. These 9 data elements were chosen as the least likely to change for an individual thus minimizing the likelihood of multiple numbers for an individual. CTCC uses the NIST/NSA designed hash function known as SHA512 because there is no known way to reverse or ‘decrypt’ the 9 data elements. It is completely a one-way function. The system then stores only the fingerprint and clears the individual’s responses from memory. The user is then assigned a number, which is associated to their fingerprint. Users never have access to the fingerprint resulting in a further level of protection. All system components are protected behind a Cisco firewall device and the individual’s responses are protected by a 128-bit secure socket layer (SSL) encryption to their point of entry.

Once the subject has signed the consent form for COHORT, the subject is directed to an accessible website (<https://www.ctcc.rochester.edu/uniqueid>) where they will enter the nine data elements as delineated above, which should not change over time. The fingerprint will be coded to a 9-digit unique identification number. This number will be provided to the subject and the coordinator will record this number on the Demographics case report form for COHORT. If at any point the subject forgets his or her unique CTCC identification number, he or she can go back to the website and enter the 9 data elements again. These 9 data elements must be identical to those entered before to obtain the same unique ID. The computer program will create the same fingerprint and return the same unique identification number.

Coriell will assign a unique repository identification number to each sample, which is different from the subject identification number and the CTCC unique identifier assigned by the CTCC**.** Blood cell DNA and DNA from the lymphoblastoid cell line sent to Massachusetts General Hospital DNA Genotyping Laboratory for genotyping will be coded and only identified by the Coriell identification number. The CTCC and Coriell will be able to link the subject identification number and the Coriell identification number, but neither Indiana nor the MGH DNA Genotyping lab will be able to link the subject number with the Coriell identification number.

All plots of family history data generated at Indiana will be created without any identifying family information (such as names). The family history data are stored on a computer running Microsoft Windows 2000 Server. This operating environment has been evaluated by the International Organization for Standardization (ISO) and meets the Common Criteria (CC) EAL 4 security rating. In addition, Windows 2000 Server provides security features over and above the EAL 4 level with the Microsoft Security Response Center. Databases are backed up daily to tape.

***Certificate of Confidentiality***

A Certificate of Confidentiality has been obtained from the Department of Health and Human Services. This certificate will be used to enhance the confidentiality for subjects in COHORT given many of the research subjects have HD or are “at-risk” for HD. The extra layer of protection is a necessity considering the potential disclosure of gene or clinical status could cause negative employment, difficulty obtaining insurance, and social or familial consequences. In addition, since subjects themselves will also remain blinded to their gene testing results, involuntary disclosure could have a negative impact on an individual’s psychological well-being and mental health.

# RISK/BENEFIT ASSESSMENT

1. **Potential Risks**

***Blood draw*** – The collection of blood specimens may cause pain and/or bruising at the site where blood is drawn. Fainting or feeling lightheaded may occur during or shortly after having blood drawn. If a subject experiences this, the subject will be instructed to lie down immediately to avoid possible injuries. Localized clot formation and infections may occur, but this is very rare.

***Unified Huntington’s Disease Rating Scale/Mini-Mental State Examination/Family History Questionnaire*** – The subject may experience anxiety or psychological discomfort while completing the UHDRS 99, MMSE, and family history questionnaire.

***Breach of Confidentiality*** – There is always the possibility of a breach of confidentiality with information that is entered into a computer database. Someone could breach the security of the computer system(s) and access information, thereby resulting in a loss of confidentiality. During the course of this study, it is possible that the investigators may develop concerns about the subject causing harm to themselves or others. In these situations, confidentiality may be breached in order to protect the subject or others.

1. **Protection Against Risks**

Blood draw/UHDRS 99/MMSE

Only experienced staff will draw the blood for this study. The UHDRS 99 and MMSE data will be labeled with only the subject identification number.

***Event Monitoring Committee***

The Event Monitoring Committee (EMC) will periodically review coded data from the COHORT study for potential safety concerns. The committee will then advise the Steering Committee about findings relevant to the conduct of the trial, and assist in training or other efforts related to human subjects issues. In the mode of a data monitoring committee, the EMC will:

1. Analyze and categorize the occurrence of pre-specified medical and mental health events;
2. Periodically advise the principal investigator and steering committee about the occurrence and significance of pre-specified medial and mental health events;
3. Develop and recommend policies related to these events;
4. Participate in or facilitate the public dissemination of the findings, via peer-reviewed publications, in concert with the Principal Investigators and Steering Committee.

The EMC may recommend that certain issues or concerns be reviewed by: 1) the Principal Investigator or Steering Committee; 2) the medical monitor; 3) the standing HSG Bioethics Advisory Committee (BAC); or 4) the standing HSG Scientific Advisory Committee (SAC).

CTCC Protections Against Breach of Confidentiality

The CTCC has several provisions in place to maintain integrity, confidentiality, and security of subject information. The CTCC computer systems and networks are managed by full-time system administrators. All (internal relational databases and programming server) network traffic is sent across a cisco switched network behind a Cisco PIX 515 Firewall. All PC’s run virus protection software full-time and are updated with the latest virus definitions and scanned on a regular schedule; the Windows servers are protected as well and we have additional security in place to scan all e-mail for viruses before a user has access to them. All servers have been customized to run the bare minimum of network services in order to minimize potential “back door” attacks, and both servers are updated on a regular basis with the latest vendor recommended software patches and updates. In addition, other security software runs continuously minimizing the potential for other threats (e.g., password “crackers” to detect easily guessable passwords). All accounts are password protected and passwords must be changed on a regular basis. All study data is stored in a relational database management system residing on one of several UNIX servers. Each study database has multiple security levels including: network access to the server, access to the database, group level access, and access to individual tables, views, and stored procedures within a database. In general, users are set up with the minimal required level of access to database objects, e.g. if a user requires read-only access to a table then he/she is set up that way. For web-based applications, SSL security is employed to protect all network traffic.

All servers reside in one of several racking systems inside a locked computer room that is physically accessible only by the departmental system administrators. The computer room is temperature controlled and has water sensors in the ceiling to alert system administrators in case of a leak from above. It is also equipped with smoke/fire detection sensors and motion sensors. To ensure high system availability the Sun server is equipped with dual power supplies. Each night the system does an “epoch” backup to SDLT tape and each private subnet.

All CRF data and other critical study data are fully audit trail enabled so that all changes to the data can be monitored and/or recovered. Permissions are carefully maintained to allow only the required level of access to study data. Files and directories are carefully set with only the required level of access.

***Indiana University Protections Against Breach of Confidentiality***

Extensive security measures have been taken to ensure the privacy and confidentiality of all data that will be sent to Indiana University. Files containing family information are housed in a secure location within the Department of Medical and Molecular Genetics at the University of Indiana. Computer files are stored on a SUN microcomputer, which is housed in a secure room within the Department of Medical and Molecular Genetics. Security of data is protected by the following authentication process: 1) the user must log in by supplying a user-name and password. Password changes are enforced on a regular basis and unsuccessful login attempts are logged and the administrator is notified.  Accounts are locked-out after several consecutive unsuccessful login attempts; 2) Access to the data is limited not only to specific users but also to the location from which they are logging-in.  A login will fail if it is not from a computer at a trusted Internet address; and 3) once the domain login is authenticated, the user must supply another database-specific password to access the data. Databases are backed-up daily to local disk and archived weekly to tape.  Use of a microcomputer for data storage has many advantages over the use of mainframe computers, the greatest of which is heightened security. In addition, the SUN operating system, SOLARIS, is a Unix system that allows for ease of data entry and query processing. Every precaution has been taken to assure that computer confidentiality is maintained.

Coriell Institute for Medical Research (Coriell Cell Repositories) Protections Against Breach of Confidentiality

The Coriell Cell Repositories has several provisions in place to maintain integrity, confidentiality, and security of its data and information systems. Coriell does not store subject’s confidential information, has security policies in place to assure that all data are protected from unauthorized access, and maintains audit trails, backup procedures and error checking to assure accuracy and protection of its data.

Protection of Subject Confidentiality Provisions

As part of the operation of its many repositories, Coriell has been proactive in maintaining the confidentiality of its donor subjects. When a sample is submitted to Coriell, it is assigned a unique repository identifier, generally a seven digit alphanumeric: the first two digits are letters indicating the repository into which it will be accessioned, and the next five digits are numbers generally beginning with 00001 assigned sequentially to each sample as it arrives at Coriell. Through all operations at Coriell, the materials are tracked using this unique identifier. There is no identification of the submitting investigator for materials that are displayed in an electronic catalog to prevent individuals from establishing the identity of the donor subject.

Coriell has its own Institutional Review Board that consists of thirteen members, six of whom work at the Institute and seven from outside the Institute. Coriell's IRB will not be utilized for this study, as the Coordination Center IRB and all of the site IRBs will review this protocol. In addition, each of the faculty members at Coriell has received Human Subjects Assurance Training and is certified through the Office for Human Research Protections.

Although Coriell is not a HIPAA covered entity, it must work directly with HIPAA covered entities both in its acquisition phase and in its distribution phase. The information in the 18 identifiers that is restricted under HIPAA is not collected at Coriell and submitters are requested not to submit such information. The lack of this information does not reduce the value of the Repositories in any way, and Coriell will continue to monitor changes in the interpretations of the HIPAA regulations.

*Network Security Provisions*

The computer systems, servers and network are maintained with full-time system administrators, and the Coriell network administrator is responsible for maintaining a secure network environment. The administrator monitors security logs on a regular basis to ensure network integrity. The administrator also manages the account credentials for all users on the network. The superuser passwords on all servers are changed every three months for additional security. The Coriell firewall is a Cisco PIX Firewall device and is configured to route traffic to a DMZ zone and an internal zone. The DMZ zone includes the servers that need to be accessed publicly, such as the web and email servers. The internal zone includes the database servers and all desktop computers. This network configuration establishes a secure environment that minimizes the risk of unauthorized access from external networks.

All desktop computers run virus protection software full-time and are updated with the latest virus detection strings regularly.

*Data Security Provisions*

The hardware platform Coriell currently uses for its collection/specimen data capture, storage, and analysis is a SUN SPARC Enterprise 450; this server is dedicated exclusively to these tasks. The SUN server resides in a racking system inside a locked server room that is physically accessible only by the Information Systems staff. The server room is equipped with smoke/fire detection sensors. To ensure high system availability the SUN server is equipped with dual power supplies, hot-swappable RAID 5 disk drives, and an APC uninterruptible power supply. The Relational Database Management Software suite is Sybase 11.9.2 (Adaptive Enterprise Server). Running concurrently with the Sybase database is the Sybase Backup Server that performs full daily backups of the Coriell databases.

Sybase contains an elaborate and comprehensive security system based on user rights and permissions. This security system is fully utilized to allow only the necessary personnel to insert or update data in the production database. For any item of data that is entered in the data repository, an audit trail record will be generated by the system.

Coriell protects against the accidental loss of valuable data by establishing documented backup and recovery procedures on a daily basis.

***Massachusetts General Hospital (MGH) DNA Genotyping Laboratory Protection Against Breach of Confidentiality***

All samples sent to MGH DNA Genotyping Laboratory for CAG genotyping and genotyping of authenticated polymorphisms in other genes previously shown to modify timing or expression of disease features will be coded. For the purposes of genotyping the lab at MGH will receive no subject data or identifying information. They will receive the blood DNA samples and the DNA from the lymphoblastoid cell line with only the Coriell Identification Number.

1. **Potential Benefit**

##### Subjects will receive no medial benefit from participation in this study. The only potential benefit is a better understanding of HD and the possibility that the information obtained in this study lead to potential treatments and to plan future research studies of experimental drugs aimed at slowing or postponing the onset of HD.

1. **Alternatives to Participation**

The only alternative to participation in this study is not to participate.

1. **Withdrawal from Participation**

Subjects may be withdrawn from the study for the following reasons:

- - - - A change in medical condition, so that remaining in the study might risk an individuals health or the research.
      - Failure to complete the required study procedures, regardless of reason.
      - The site investigator feels that it is in the best interest of the subject.

# STATISTICAL CONSIDERATIONS

1. **Sample Size Calculation**

Prior to the initiation of studies to identify the genetic determinants of a disease phenotypic, it is essential to first estimate that fraction of the phenotypic variation due to genetic factors. A commonly employed design to perform such an analysis is the collection of sibling pairs with the goal of estimating the heritability of the phenotype of interest. The COHORT sample will recruit multiple members from a family and through the collection of family history information, will be able to establish the biological relationship among individuals and use this information for genetic analyses. Siblings are typically employed for heritability studies since they are closely related and are expected to share, on average, half their genetic material. Studies in COHORT will focus on estimating the heritability of a number of different quantitative phenotypes including the age of onset of disease and the rate of disease progression (as measured by a number of different quantitative traits). In addition, with the collection of a number of novel biomarkers, COHORT is uniquely positioned to estimate the heritability of these biomarkers to determine whether genes are important predictors of their variability. For those phenotypes and traits, which are heritable, the collection of family data as part of COHORT will allow us to initiate studies to identify the genes and polymorphisms contributing to trait variability.

Broad-sense heritability (H^2^) is estimated as twice the sibling intraclass correlation, according to the method of Falconer (1989).  As shown below, for a phenotypic trait with 50% heritability (i.e. half the phenotypic variation is due to genetic effects), a sample of approximately 100 sibling pairs will have 80% power (with alpha=0.05). As the genetic contribution to the trait decreases, the required sample size increases. For a trait with only 20% heritability, a sample of approximately 600 sibling pairs is required for 80% power.

Falconer DS. 1989. *Introduction to Quantitative Genetics.* 3rd ed. Essex, England: Longman Scientific and Technical.

The COHORT study was designed to assemble a large descriptive database that will provide the opportunity to study many different factors potentially associated with HD-related events. Some potential factors include history of smoking, current concomitant medication use, or an unknown gene possibly associated with a predisposition to HD. The primary outcome for those affected with HD is progression of disease, which is measured by a decrease in total functional capacity (captured on the UHDRS 99). The primary outcome for those at risk would be phenoconversion or clinical onset of disease.

The table below gives the numbers of events required to detect an increase in risk corresponding to a hazard ratio of the specified magnitude associated with a single risk factor with the specified prevalence. A two-sided alpha level of 5% is assumed. The numbers of events given are for 80% power; for 90% power multiply all entries by 1.34.

| **Risk Factor Prevalence** | **Hazard Ratio** | | | | | | | |
| --- | --- | --- | --- | --- | --- | --- | --- | --- |
|  | **5.0** | **4.0** | **3.0** | **2.5** | **2.0** | **1.8** | **1.6** | **1.5** |
| **1%** | 306 | 412 | 656 | 943 | --- | --- | --- | --- |
| **2%** | ***154*** | ***208*** | 331 | 476 | 833 | --- | --- | --- |
| **3%** | ***104*** | ***140*** | 223 | 321 | 561 | 780 | --- | --- |
| **4%** | ***79*** | ***106*** | ***169*** | 243 | 425 | 591 | 924 | --- |
| **5%** | ***64*** | ***86*** | ***137*** | ***197*** | 344 | 478 | 747 | --- |
| **6%** | ***54*** | ***72*** | ***115*** | ***166*** | 289 | 402 | 629 | 846 |
| **8%** | ***41*** | ***55*** | ***88*** | ***127*** | 222 | 308 | 482 | 648 |
| **10%** | ***34*** | ***45*** | ***72*** | ***104*** | ***181*** | 252 | 394 | 530 |
| **12%** | ***29*** | ***39*** | ***62*** | ***88*** | ***155*** | ***215*** | 336 | 452 |
| **14%** | ***25*** | ***34*** | ***54*** | ***78*** | ***136*** | ***188*** | 295 | 396 |
| **16%** | ***23*** | ***30*** | ***48*** | ***69*** | ***121*** | ***169*** | 264 | 355 |
| **18%** | ***21*** | ***28*** | ***44*** | ***63*** | ***111*** | ***154*** | 240 | 323 |
| **20%** | ***19*** | ***25*** | ***41*** | ***58*** | ***102*** | ***142*** | 222 | 298 |
| **25%** | ***16*** | ***22*** | ***35*** | ***50*** | ***87*** | ***121*** | ***189*** | 254 |
| **30%** | ***14*** | ***19*** | ***31*** | ***44*** | ***78*** | ***108*** | ***169*** | 227 |
| **35%** | ***13*** | ***18*** | ***29*** | ***41*** | ***72*** | ***100*** | ***156*** | ***210*** |
| **40%** | ***13*** | ***17*** | ***27*** | ***39*** | ***68*** | ***95*** | ***148*** | ***199*** |
| **45%** | ***12*** | ***16*** | ***26*** | ***38*** | ***66*** | ***92*** | ***143*** | ***193*** |
| **50%** | ***12*** | ***16*** | ***26*** | ***37*** | ***65*** | ***91*** | ***142*** | ***191*** |

Suppose we are interested in a risk factor with a prevalence of 10%. We would like to know how much greater the risk of a relevant phenotypic event (e.g., progression of HD or phenoconversion) is for those with the risk factor compared to those without the risk factor. If we want to be 80% sure to detect a hazard ratio of at least 2.0 (e.g., double the risk), we would need 181 subjects to reach that event (e.g., phenoconvert). If we want to be 90% sure, we would need 181 X 1.34=243 subjects to reach that event.

The above hazard ratio table shows that if 220 subjects phenoconvert, we will have good power to detect substantial effects (hazard ratio exceeding 2.0 –) of the risk factor assuming that is they are not too rare in the study population (bold). For example, assuming that history of smoking is prevalent in the study population, we should be able to detect the increased risk in phenoconversion due to history of smoking.

Only those subjects in COHORT who are unaffected HD gene carriers are at risk to phenoconvert. In theory, this population will include all unaffected subjects who are known to carry the HD gene (as a result of pre-symptomatic DNA testing) and about 40% of the unaffected subjects with a HD parent who do not know their gene status (chose not to undergo DNA testing) but are expected by probability to carry the HD gene. As more of these subjects are enrolled and followed to phenoconversion, COHORT will have increasing power to detect genetic polymorphisms or other factors that influence the clinical onset of HD.

The NIH-sponsored prospective studies “Neurobiological Predictors of Huntington’s Disease” (Predict-HD - that is examining phenotype among unaffected subjects who are known to carry the HD gene as a result of presymptomatic DNA testing), and “Prospective Huntington’s at Risk Observational Study (PHAROS - that is examining phenotype among unaffected subjects with a HD parent who have unknown gene status having chosen not to undergo DNA testing) will in the coming years provide increasingly accurate estimates of phenoconversion, including the sensitivity and specificity of this therapeutically relevant phenotype. COHORT will also provide an ongoing observational study for interested and eligible subjects who were not able to participate in Predict-HD or PHAROS as well as for subjects who conclude their research participation in Predict-HD and PHAROS.

**b) Data Analysis**

Statistical and analytical methods will be determined by the Huntington Study Group (HSG) with guidance from Biostatistics at the University of Rochester and carried out through the Clinical Trials Coordination Center of the Movement Disorders Unit, Department of Neurology, University of Rochester.

## CONSULTANTS

1. **Planning Committee**

- Robi Blumenstein
- Michael Conneally
- Tatiana Foroud
- Jacqueline Gray
- James Gusella
- Madeline Harrison
- Elise Kayson
- Karl Kieburtz
- Martha Nance
- David Oakes
- Kelley O’Donoghue
- Megan Romer
- Christopher Ross
- Aileen Shinaman
- Ira Shoulson
- Oksana Suchowersky
- Terry Tempkin
- Alan Tobin

1. **Steering Committee**

- Robi Blumenstein *(ex-officio)*
- Michael Conneally
- Tatiana Foroud (co-PI)
- Jacqueline Gray *(ex-officio)*
- James Gusella (co-PI)
- Madaline Harrison
- Elise Kayson *(ex officio)*
- Karl Kieburtz *(ex-officio)*
- Richard Myers
- Martha Nance
- David Oakes
- Kelley O’Donoghue *(ex-officio)*
- Aileen Shinaman (Executive Director, *ex-officio)*
- Ira Shoulson (PI)
- Julie Stout
- Oksana Suchowersky
- Terry Tempkin

# X. References

1. Kowall N, Ferrante R, Martin J. Patterns of cell loss in Huntington's disease. Trends in Neuroscience 1987;10:24-29. [↑](#endnote-ref-2)
2. Riley D, Lang A. Movement Disorders. In: Bradley W, Daroff R, Fenichel G, CD M, editors. Neurology in Clinical Practice, Vol. II. The Neurological Disorders. Boston: Butterworth-Heinemann; 1991. p. 1563-1601. [↑](#endnote-ref-3)
3. Adams P, Falek A, Arnold A. Huntington's disease in Georgia: age at onset. J Hum Genet 1988;43:695-704. [↑](#endnote-ref-4)
4. Oliver J. Huntington's chorea in Northamptonshire. Br J Psychiat 1970;116:241-253. [↑](#endnote-ref-5)
5. Conneally P. Huntington's disease: genetics and epidemiology. Am J Hum Genet 1984;36:506-526. [↑](#endnote-ref-6)
6. Harper P. The epidemiology of Huntington's disease. Human Genetics 1992;89:365-376. [↑](#endnote-ref-7)
7. Tanner C, Goldman S. Epidemiology of movement disorders. Current Opinion in Neurology 1994;7:325-332. [↑](#endnote-ref-8)
8. Quaid K, Morris M. Reluctance to undergo predictive testing: the case of Huntington's disease. Am J Med Genet 1993;45:41-45. [↑](#endnote-ref-9)
9. Young A, Shoulson I, Penney J, Starosta-Rubinstein S, Gomez F, Travers H, et al. HD in Venezuela: Neurologic features and functional decline. Neurology 1986;36:244-249. [↑](#endnote-ref-10)
10. Bruyn G. Huntington's chorea: historical clinical and laboratory synopsis. In: Vinken P, Bruyn G, editors. Handbook of Clinical Neurology. Amsterdam, North Holland; 1968. p. 298-378. [↑](#endnote-ref-11)
11. Leigh R, Newman S, Folstein S, Lasker A. Abnormal ocular motor control in Huntington's disease. Neurology 1983;33:1268-1275. [↑](#endnote-ref-12)
12. Collewijn H, Went L, Tomminga E, Vegter-van de Vlis M. Oculomotor defects in patients with Huntington's disease and their offspring. J Neurol Sci 1988;86:307-320. [↑](#endnote-ref-13)
13. Young A, Shoulson I, Penney J, Starosta S, Gomez F, Travers H, et al. Huntington's disease in Venezuela: neurologic features and functional decline. Neurology 1986;36:244-249. [↑](#endnote-ref-14)
14. Caine E, Hunt R, Weingartner H, Ebert M. Huntington's dementia: clinical and neuropsychological features. Arch Gen Psychiat 1978;35:377-384. [↑](#endnote-ref-15)
15. Bamford K, Caine E, Kido D, Plassche W, Shoulson I. Clinical-pathologic correlation in Huntington's disease: A neuropsychological and computed tomography study. Neurology 1989;39:796-801. [↑](#endnote-ref-16)
16. Caine E, Shoulson I. Psychiatric syndromes in Huntington's disease. Am J Psychiat 1983;140:728-733. [↑](#endnote-ref-17)
17. Folstein S, Chase G, Wahl W, McDonnell A, Folstein M. Huntington's disease in Maryland: clinical aspects of racial variation. Am J Hum Genet 1987;41:168-171. [↑](#endnote-ref-18)
18. Shoulson I, Fahn S. Huntington's disease: clinical care and evaluation. Neurology 1979;29(1-3). [↑](#endnote-ref-19)
19. Feigin A, Kieburtz K, Bordwell K, Como P, Steinberg K, Sotack J, et al. Functional decline in Huntington's disease. Movement Disorders 1995;10(2):211-214. [↑](#endnote-ref-20)
20. Myers R, Sax D, Koroshetz W, Mastromauro C, Cupples L, Kiely D, et al. Factors associated with slow progression in Huntington's disease. Arch Neurol 1991;48:800-804. [↑](#endnote-ref-21)
21. Penney J, Young A, Shoulson I, Starosta-Rubenstein S, Snodgrass S, Sanchez Ramos J, et al. Huntington's disease in Venezuela: 7 years of follow-up on symptomatic and asymptomatic individuals. Movement Disorders 1990;5:93-99. [↑](#endnote-ref-22)
22. Shoulson I, Odoroff C, Oakes D, Behr J, Goldblatt D, Caine E, et al. A controlled clinical trial of baclofen as protective therapy in early Huntington's disease. Ann Neurol 1989;25:252-259. [↑](#endnote-ref-23)
23. Young A, Penney J, Starosta-Rubinstein S, Markel D, Berent S, Giordani B, et al. PET scan investigations of Huntington's disease: cerebral metabolic correlates of neurological features and functional decline. Ann Neurol 1986;20:296-303. [↑](#endnote-ref-24)
24. Kido D, Shoulson I, Manzione J, Harnish P. Measurement of caudate nucleus and putamen atrophy in patients with Huntington's disease. Neuroradiology 1991;33:604-606. [↑](#endnote-ref-25)
25. Mazziota J. Huntington's disease: studies with structural imaging techniques and positron emission tomography. Semin Neurol 1994;9(4):360-369. [↑](#endnote-ref-26)
26. Vonsattel J, Myers R, Stevens T, Ferrante R, Bird E, Richardson EJ. Neuropathological classification of Huntington's disease. J Neuropath Exp Neurol 1985;44:559-577. [↑](#endnote-ref-27)
27. Jenkins B, Koroshetz W, Beal F, Rosen B. Evidence for impairment of energy metabolism in vivo in Huntington's disease using localize H NMR spectroscopy. Neurology. 1993;43:2689-2695. [↑](#endnote-ref-28)
28. The Huntington Study Group (Karl Kieburtz). Unified Huntington's Disease Rating Scale: reliability and consistency. Movement Disorders. 1996;11:136-142. [↑](#endnote-ref-29)
29. The Huntington Study Group (Peter Como) Unified Huntington's Disease Rating Scale (abstract). Neurology. 1995;45(4):254. [↑](#endnote-ref-30)
30. The Huntington Study Group (Karen Marder). Rate of functional decline in Huntington's disease. Neurology. 2000; 54(2):452-458. [↑](#endnote-ref-31)
31. The Huntington Study Group (Frederick Marshall), Safety and tolerability of the free-radical scavenger OPC-14117 in Huntington's disease. Neurology, 1998. 50: p. 1366-1373. [↑](#endnote-ref-32)
32. Kieburtz K, Feigin A, McDermott M, Como P, Abwender D, Zimmerman C, Hickey C, Orme C, Claude K, Sotack J, Greenamyre J, Dunn C, Shoulson I. A controlled trial of remacemide hydrochloride in Huntington's disease. Movement Disorders. 1996;11:272-277. [↑](#endnote-ref-33)
33. The Huntington Study Group (Karl Kieburtz). Coenzyme Q10 and Remacemide Evaluation in Huntington's disease (CARE-HD). 17th International Meeting of the World Federation of Neurology Research Group on Huntington's Disease Program and Abstracts. Sydney; 1997: (330 August - 2 September, 1997), p. 33. [↑](#endnote-ref-34)
34. Feigin A, Kieburtz K, Como P, Abwender D, Zimmerman C, Hickey C, Steinberg K, Bordwell K, Sotack J, Orme C, Shoulson I. An open-label trial of coenzyme Q10 (CoQ) in Huntington's disease (HD). Neurology. 1994;44(2):A397-A398. [↑](#endnote-ref-35)
35. Benton A, Hamsher K. Multilingual Aphasia Examination Manual. Iowa City: University of Iowa; 1978. [↑](#endnote-ref-36)
36. Smith A. Symbol Digit Modalities Test Manual. Los Angeles: Western Psychological Services; 1973. [↑](#endnote-ref-37)
37. Stroop J. Studies of interference in serial verbal reactions. J Exper Psychol. 1935;18:643-662. [↑](#endnote-ref-38)
38. Bylsma F, Como P, Paulsen J, Jones R, Rey G, Saint-Syr J, Stebbins G, Group HS. Noncognitive symptoms in Huntington's disease. JINS 1996; 2:35. [↑](#endnote-ref-39)
39. Shoulson I, Kurlan R, Rubin A, Goldblatt D, Behr J, Miller C, Kennedy J, Bamford K, Caine E, Kido D, Plumb S, Odoroff C. Assessment of functional capacity in neurodegenerative movement disorders: Huntington's disease as a prototype. In: Munsat T, ed. Quantification of Neurological Deficit. Boston: Butterworths; 1989:271-283. [↑](#endnote-ref-40)
40. Folstein MF, Folstein SE, McHugh PR. Mini-Mental State: A practical method for grading the cognitive state of patients for the clinician. J Psychiatric Res 1975;12:189-198. [↑](#endnote-ref-41)
41. Huntington's Disease Collaborative Research Group (HDCRG) A novel gene containing a trinucleotide repeat that is expanded and unstable on Huntington's disease chromosomes. Cell 1993;72:971-983. [↑](#endnote-ref-42)
42. Andrew SE, Goldberg YP, Kremer B, Telenius H, Theilmann J, Adam S, Starr E, Squitieri F, Lin B, Kalchman MA, Graham RK, Hayden MR. The relationship between trinucleotide (CAG) repeat length and clinical features of Huntington's disease. Nat Genet 1993;4:398-403. [↑](#endnote-ref-43)
43. Duyao M, Ambrose C, Myers R, Novelletto A, Persichetti F, Frontali M, Folstein S, et al. Trinucleotide repeat length instability and age of onset in Huntington's disease. Nat Genet 1993;4:387-392. [↑](#endnote-ref-44)
44. Snell RG, MacMillan JC, Cheadle JP, Fenton I, Lazarou LP, Davies P, MacDonald ME, Gusella JF, Harper PS, Shaw DJ. Relationship between trinucleotide repeat expansion and phenotypic variation in Huntington's disease. Nat Genet 1993;4:393-397. [↑](#endnote-ref-45)
45. Ranen NG, Stine OC, Abbott MH, Sherr M, Codori AM, Franz ML, Chao NI, Chung AS, Pleasant N, Callahan C, Kasch LM, Ghaffari M, Chase GA, Kazazian HH, Brandt J, Folstein SE, Ross CA. Anticipation and instability of IT-15 (CAG)n repeats in parent-offspring pairs with Huntington disease. Am J Hum Genet 1995;57:593-602. [↑](#endnote-ref-46)
46. Brinkman RR, Mezei MM, Theilmann J, Almqvist E, Hayden MR. The likelihood of being affected with Huntington disease by a particular age, for a specific CAG size. Am J Hum Genet 1997;60:1202-1210. [↑](#endnote-ref-47)
47. Djoussé L, Knowlton B, Hayden M, Almqvist EW, Brinkman R, Ross C, Margolis R, et al. Interaction of normal and expanded CAG repeat sizes influences age at onset of Huntington disease. Am J Med Genet 2003;119A:279-282. [↑](#endnote-ref-48)
48. Wexler NS, Lorimer J, Porter J, Gomez F, Moskowitz C, Shackell E, Marder K, Penchaszadeh G, Roberts SA, Gayan J, Brocklebank D, Cherny SS, Cardon LR, Gray J, Dlouhy SR, Wiktorski S, Hodes ME, Conneally PM, Penney JB, Gusella J, Cha JH, Irizarry M, Rosas D, Hersch S, Hollingsworth Z, MacDonald M, Young AB, Andresen JM, Housman DE, De Young MM, Bonilla E, Stillings T, Negrette A, Snodgrass SR, Martinez-Jaurrieta MD, Ramos-Arroyo MA, Bickham J, Ramos JS, Marshall F, Shoulson I, Rey GJ, Feigin A, Arnheim N, Acevedo-Cruz A, Acosta L, Alvir J, Fischbeck K, Thompson LM, Young A, Dure L, O’Brien CJ, Paulsen J, Brickman A, Krch D, Peery S, Hogarth P, Higgins DS Jr, Landwehrmeyer B; U.S.-Venezuela Collaborative Research Project. Related Articles, Links Venezuelan kindreds reveal that genetic and environmental factors modulate Huntington’s disease age of onset. Proc Natl Acad Sci U S A. 2004 Mar 9;101(10):3498-503. [↑](#endnote-ref-49)
49. Naze P, Vuillaume I, Destee A, Pasquier F, Sablonniere B. Mutation analysis and association studies of the ubiquitin carboxy-terminal hydrolase L1 gene in Huntington's disease. Neurosci Lett 2002;328-14. [↑](#endnote-ref-50)
50. Rubinsztein DC, Leggo J, Chiano M, Dodge A, Norbury G, Rosser E, Craufurd D. Genotypes at the GluR6 kainate receptor locus are associated with variation in the age of onset of Huntington disease. Proc Natl Acad Sci USA 1997;94:3872-3876. [↑](#endnote-ref-51)
51. MacDonald ME, Vonsattel JP, Shrinidhi J, Couropmitree NN, Cupples LA, Bird ED, Gusella JF, Myers RH. Evidence for the GluR6 gene associated with younger onset age of Huntington's disease. Neurology 1999;53:1330-1332. [↑](#endnote-ref-52)
52. Li JL, Hayden MR, Almqvist EW, Brinkman RR, Durr A, Dodé C, Morrison PJ, Suchowersky O, Ross CA, Margolis RL, Rosenblatt A, Gómez-Tortosa E, Cabrero DM, Novelletto A, Frontali M, Nance M, Trent RJA, McCusker E, Jones R, Paulsen JS, Harrison M, Zanko A, Abramson RK, Russ AL, Knowlton B, Djoussé L, Mysore JS, Tariot S, Gusella MF, Wheeler VC, Atwood LD, Cupples LA, Saint-Hilaire M, Cha JJ, Hersch SM, Koroshetz WJ, Gusella JF, MacDonald ME, and Myers RH. A Genome Scan for Modifiers of Age at Onset in Huntington Disease: A HD MAPS Study. Am J Hum Genet 2003; 73:682-687.

    **Appendix**

    *(Includes other associated protocol documents)*

    Family History Questionnaire (revised 8/31/06)

    Family History Update (revised 8/31/06)

    COHORT Data & Specimen Repository Access Policy and Procedures

    [↑](#endnote-ref-53)
